# Near-Field Channel Parameter Estimation and Localization for mmWave Massive MIMO-OFDM ISAC Systems via Tensor Analysis

**DOI:** 10.3390/s25165050

**Published:** 2025-08-14

**Authors:** Lanxiang Jiang, Jingyi Guan, Jianhe Du, Wei Jiang, Yuan Cheng

**Affiliations:** School of Information and Communication Engineering, Communication University of China, Beijing 100024, China; lovejianglx@cuc.edu.cn (L.J.); dujianhe1@gmail.com (J.D.); jw@cuc.edu.cn (W.J.); chengyuan2018@cuc.edu.cn (Y.C.)

**Keywords:** near-field, tensor, parameter estimation, localization, Cramér–Rao Bound

## Abstract

Integrated Sensing And Communication (ISAC) has been applied to the Internet of Things (IoT) network as a promising 6G technology due to its ability to enhance spectrum utilization and reduce resource consumption, making it ideal for high-precision sensing applications. However, while the introduction of millimeter Wave (mmWave) and massive Multiple-Input Multiple-Output (MIMO) technologies can enhance the performance of ISAC systems, they extend the near-field region, rendering traditional channel parameter estimation algorithms ineffective due to the spherical wavefront channel model. Aiming to address the challenge, we propose a tensor-based channel parameter estimation and localization algorithm for the near-field mmWave massive MIMO-Orthogonal Frequency Division Multiplexing (OFDM) ISAC systems. Firstly, the received signal at the User Terminal (UT) is constructed as a third-order tensor to retain the multi-dimensional features of the data. Then, the proposed tensor-based algorithm achieves the channel parameter estimation and target localization by exploiting the second-order Taylor expansion and intrinsic structure of tensor factor matrices. Furthermore, the Cramér–Rao Bounds (CRBs) of channel parameters and position are derived to establish the lower bound of errors. Simulation results show that the proposed tensor-based algorithm is superior compared to the existing algorithms in terms of channel parameter estimation and localization accuracy in ISAC systems for IoT network, achieving errors that approach the CRBs. Specifically, the proposed algorithm attains a 79.8% improvement in UT positioning accuracy compared to suboptimal methods at SNR = 5 dB.

## 1. Introduction

### 1.1. Background

With the advancement of numerous next-generation communication technologies, the demand for communication services and sensing has surged, which makes it extremely necessary to find effective methods to improve the spectral efficiency [1,2]. Integrated Sensing And Communication (ISAC), as a key technology in Sixth Generation (6G) networks, has attracted extensive attention from both the industry and the academia due to its ability to alleviate the congestion of existing spectrum [3]. In addition, the separated design of sensing and communication in traditional communication systems cannot meet the requirements of applications for the high transmission rate and the sensing accuracy. The ISAC technology can achieve information sharing in sensing and communication, thereby optimizing the joint design of parameters and architectures, which is conducive to improving the overall performance of the system and saving hardware resources [4,5]. In traditional communication systems, significant differences in hardware architectures exist between communication and sensing functions, caused by their different service requirements, algorithm designs and evaluation metrics. In ISAC system design, hardware resource sharing is regarded as a critical component [6], with most hardware resources being shared between communication and sensing through carefully balancing ISAC performance and resources utilization efficiency. For the ISAC architecture, the same transmitted signal is not only used for the data transmission, but also for the target sensing, tracking and navigation [7]. Generally speaking, high-frequency signals enable high-resolution sensing through narrow beams, while the communication function leverages sensing parameters (e.g., target position, channel state) to optimize the beam direction and the resource allocation. Specifically, by taking advantage of the reflection and scattering characteristics of electromagnetic waves, the sensing can be applied to obtain environmental information for performing tasks such as positioning [8] and environmental reconstruction [9]. Therefore, the sensing plays an extremely important role in communication systems and has become a research hotspot.

The Internet of Things (IoT) has been considered an effective solutions in terms of intelligent internet and social problems [10]. To meet the increasing demand for enhanced communication and sensing performance of future IoT networks, the massive Multiple-Input Multiple-Output (MIMO) and millimeter Wave (mmWave) technology have been widely studied for ISAC systems, enabling higher communication capacity and improved spatial and temporal resolution [11,12]. In addition to providing abundant spectrum resources, the short wavelength of mmWaves allows for reduced antenna spacing, making it easier to encapsulate antenna arrays into small devices, which can generate high directional beamforming and reduce hardware costs [13]. The introduction of MIMO technology can be exploited to enhance the spectral efficiency and the beamforming gain to resist the high-frequency signal degradation caused by atmospheric absorption and material penetration, thereby meeting the low-latency and high-reliability communication requirements of 6G systems [14]. Moreover, the narrow-beam characteristics of antenna arrays further augment range resolution and facilitate precise localization capabilities [15]. However, the frequency-selective fading caused by multipath effects and the ultra-large bandwidth of mmWave communication renders the narrowband transmission design based on flat fading channels unable to be directly applied to mmWave systems [16]. To better exert the advantages of the mmWave frequency band, Orthogonal Frequency Division Multiplexing (OFDM) technology based on hybrid precoding has been widely studied to resist the frequency-selective fading [17].

### 1.2. Preliminaries

To enhance the readability of the article and make its content complete, the following is a brief introduction to the basic knowledge of tensors. For more details of tensors, please refer to [18]. Tensors are higher-order generalizations of scalars, vectors and matrices; that is to say, vectors and matrices are special tensors with one and two orders or modes, respectively. An *N*-th order complex-valued tensor $X\in C^{I_{1}\times I_{2}\times \ldots \times I_{N}}$ is defined over *N* modes, with $I_{n}$ denoting the cardinality of the *n*-th mode and its $\left(i_{1},i_{2},\ldots ,i_{N}\right)$-th entry denoted by $X_{i_{1},i_{2},\ldots ,i_{N}}$. The mode-*n* vector of X is an $I_{n}$-dimensional vector with $i_{n}$ as the element subscript variable, and all other subscripts $\left\{i_{1},\ldots ,i_{N}\right\}\setminus i_{n}$ are fixed and unchanging, which is denoted by the vector $X_{i_{1}\ldots i_{n-1}:i_{n+1}\ldots i_{N}}$. A tensor slice is a matrix formed by holding all indices constant except those corresponding to two specified dimensions $n_{1}$ and $n_{2}$, i.e., $X_{i_{1},\ldots ,i_{n_{1}-1},:,:,i_{n_{1}+2},\ldots ,i_{N}}\in C^{I_{n_{1}}\times I_{n_{2}}}$. The matrix transformation of tensors is sometimes also referred to as the unfolding or flattening of tensors. The matricization operation along mode-*n*, represented as ${\left[X\right]}_{\left(n\right)}$, converts tensor X into a matrix whose columns consist of the mode-*n* fibers of X. The *n*-mode product of X with a matrix $A\in C^{L\times I_{n}}$, defined by $X{\times \;}_{n}A$, is a tensor of size $I_{1}\times \ldots \times I_{n-1}\times L\times I_{n+1}\times \ldots \times I_{N}$. Accordingly, the general Tucker decomposition can be expressed as

(1) $$X=G\times_{1}A^{\left(1\right)}\times_{2}A^{\left(2\right)}\times_{3}\ldots \times_{N}A^{\left(N\right)},$$

where $A^{\left(n\right)}\in C^{I_{n}\times L_{n}}\left(n=1,\ldots ,N\right)$ is the factor matrix of the *n*-th mode, and $G\in C^{J_{1}\times J_{2}\times \ldots \times J_{N}}$ is the core tensor. Beyond Tucker decomposition, Canonical Polyadic (CP) decomposition represents another fundamental tensor factorization approach, and it can be viewed as a constrained Tucker decomposition with a super-diagonal identity core tensor, i.e.,

(2) $$X=I_{N,L_{1}\times L_{2}\times \ldots \times L_{N}}\times_{1}A^{\left(1\right)}\times_{2}A^{\left(2\right)}\times_{3}\ldots \times_{N}A^{\left(N\right)},$$

where $I_{N,L_{1}\times L_{2}\times \ldots \times L_{N}}$ is an identity tensor with all 1s on the super-diagonal. Note that below we will briefly introduce CP decomposition by taking the third-order tensor $Y\in C^{I\times J\times K}$ as an example. If the tensor Y can be represented as the outer product of three vectors, it is a rank-1 tensor; specifically,

(3) $$Y=u^{\left(1\right)}\circ u^{\left(2\right)}\circ u^{\left(3\right)},$$

where $u^{\left(1\right)}\in C^{I\times 1}$, $u^{\left(2\right)}\in C^{J\times 1}$ and $u^{\left(3\right)}\in C^{K\times 1}$. More generally, the CP decomposition represents an arbitrary tensor as a sum of rank-1 components. For a tensor Y of rank *R*, its CP decomposition is given by

(4) $$Y=\sum_{r=1}^{R}u_{r}^{\left(1\right)}\circ u_{r}^{\left(2\right)}\circ u_{r}^{\left(3\right)}.$$

Note that the CP rank *R* of the tensor Y is expressed as rank(Y). For a given factor matrix $U^{\left(n\right)}$, its Kruskal rank $k(U^{\left(n\right)})$ corresponds to the largest integer where any subset of $k(U^{\left(n\right)})$ column vectors exhibits full column rank. By definition, this cannot exceed the matrix’s conventional rank, i.e., $k\left(U^{\left(n\right)}\right)\leq \mathrm{rank}\left(U^{\left(n\right)}\right)$. Based on the frontal slice $Y_{::k}\in C^{I\times J}$ of tensor Y, there are three different modes unfolded as follows:

(5) $$\begin{array}{cc} {\left[Y\right]}_{\left(1\right)} & ={\left[Y_{::1},\ldots ,Y_{::K}\right]}^{T}=\left(U^{\left(3\right)}⊙U^{\left(2\right)}\right){U^{\left(1\right)}}^{T}\in C^{JK\times I},\\ {\left[Y\right]}_{\left(2\right)} & ={\left[Y_{::1}^{T},\ldots ,Y_{::K}^{T}\right]}^{T}=\left(U^{\left(3\right)}⊙U^{\left(1\right)}\right){U^{\left(2\right)}}^{T}\in C^{IK\times J},\\ {\left[Y\right]}_{\left(3\right)} & =\left[\mathrm{vec}\left(Y_{::1}\right),\ldots ,\mathrm{vec}\left(Y_{::K}\right)\right]=\left(U^{\left(2\right)}⊙U^{\left(1\right)}\right){U^{\left(3\right)}}^{T}\in C^{IJ\times K}. \end{array}$$

### 1.3. Organization

The remaining content of this paper is arranged as follows. Section 2 introduces related works. Contributions and notation meanings of the article are introduced in Section 3. In Section 4, the downlink ISAC system model and the second-order Taylor expansion process are introduced. Then, the proposed tensor-based algorithm is presented in Section 5. In Section 6, the uniqueness of tensor decomposition and the complexity of algorithm are analyzed. In Section 7, simulation experiments are carried out to verify the effectiveness and robustness of the proposed algorithm for ISAC. Finally, we conclude this paper in Section 8.

## 2. Related Works

The channel estimation, as an important component in the ISAC system, can provide Channel State Information (CSI) to assist in adjusting the resource allocation of the Base Stations (BSs) and the modulation mode, thereby improving the spectral efficiency and the transmission rate. The application of Deep Learning (DL), a major Artificial Intelligence (AI) advancement, has demonstrated remarkable success in improving channel estimation for signal processing applications. By taking advantage of the inherent characteristics of the sensing channel, the work in [19] developed a diffusion neural network, transforming the channel estimation problem into a signal denoising task to improve the sensing performance in the ISAC system. The authors of [20] introduced a model-driven ISAC framework where DL-based hyperparameters enhance iterative optimization algorithms, enabling the simultaneous channel reconstruction and the target perception. However, compared with traditional channel estimation algorithms, AI-based algorithms require huge amounts of training data, which significantly increases the computational load and processing time. In light of these limitations, the authors in [21] designed a reduced MIMO channel estimation scheme for wideband communication in mmWave systems by using low-complexity antenna selection. In ref. [22], a novel measurement matrix based on time-domain correlation was constructed, along with a corresponding channel estimation algorithm designed for a specific frame structure. By utilizing the low-rank structure caused by angular spreads and sparse characteristics of the mmWave channel, a two-stage compressed sensing algorithm in [23] was proposed to estimate the mmWave channel. Furthermore, the channel estimation can be used to extract parameters such as Angles of Arrival (AoAs), Angles of Departure (AoDs) and Time of Arrival (ToA), for application in sensor networks and the target localization. Unlike references [21,22,23], which only estimate the channel without considering the parameter extraction, the authors in [24] developed a joint estimation algorithm based on the coherent time to extract the angles, distance and velocity information of the target, enhancing the sensing capability at the BS for the MIMO-OFDM ISAC system. Xia et al. in [25] proposed a two-stage estimation scheme that jointly estimates the CSI and AoD based on the channel feature projection and the angle domain subspace pruning, adapting to the dynamic changes in the communication environment in the ISAC system. A two-part coupled Multiple Signal Classification (MUSIC) algorithm with low-complexity in [26] was developed to estimate AoD and ToA, and verified that the proposed time–frequency dual extension framework has more advantages under the specific array configurations with motion.

However, the expansion of the near-field region driven by the increased number of antennas and carrier frequencies makes near-field communication investigation non-negligible [27]. Although the above works solved the parameter estimation problem in the far-field, these algorithms cannot be directly applied to the near-field model with spherical wavefront curvature. With the increase in User Terminals (UTs) and application categories in IoT networks, this issue is urgently needed to be addressed for enhancing the reliability of communication links and meeting the quality of service. Therefore, it is necessary to study the parameter estimation algorithm for the near-field to satisfy the continuous growth of future ISAC system with high-frequency bands and large-scale antennas [28]. Li et al. in [29] proposed a matrix pencil-based algorithm combining the spherical wavefront reformulation with time–frequency domain mapping to address the near-field channel estimation problem. In ref. [30], an efficient sequential angle-distance estimation algorithm was developed for near-field massive MIMO systems. Taking advantage of a distance-parameterized angular-domain sparse model, the authors in [31] proposed a joint dictionary learning and sparse recovery-based algorithm to estimate channel parameters in the near-field. Meanwhile, the simultaneous channel estimation and localization can be regarded as a critical component of ISAC, which remains essential even in the near-field. In ref. [32], a near-field uplink channel estimation scheme was proposed that leverages power sensors embedded in the antenna array to enhance sensing performance while reducing baseband sampling requirements and dictionary size. Additionally, they proposed a time-reversal-based algorithm for precise target localization. By employing the oblique projection operator, the authors in [33] developed a low-complexity one-dimensional iterative algorithm to estimate the AoD and the positions of near-field sources.

The works [29,30,31,32,33] have achieved channel estimation in the near-field, whereas, these matrix-based algorithms did not consider the multi-dimensional nature of the received signal. In recent years, tensors have been widely used in signal processing due to their high-dimensional structure. For instance, Zhang et al. in [34] formulated the frequency-domain channel as a tensor and proposed a tensor decomposition-based algorithm for the mmWave massive MIMO channel parameter estimation. The authors in [35] constructed the received signal into a third-order tensor model and proposed a channel estimation algorithm combining the Nelder–Mead simplex and the two-dimensional correlation search method for the mmWave MIMO-OFDM system. A tensor train decomposition method in [36] was introduced for signal subspace estimation, followed by employing an iterative algorithm to achieve higher parameter estimation precision. Lin et al. in [37] proposed a structured CP decomposition algorithm by using tensor theory and spatial smoothing techniques to estimate channel parameters. In ref. [38], a microstrip-sequential channel training method was proposed, representing the received frequency-domain training signal as a fourth-order tensor and enabling a non-iterative algorithm to estimate factor matrices and extract physical parameters. Nevertheless, the algorithms in [34,35,36,37,38] failed to address the near-field channel parameter estimation problem.

## 3. Contributions and Notations

### 3.1. Contributions

Inspired by the above, we propose a third-order tensor-based channel parameter estimation and localization algorithm for the near-field mmWave massive MIMO-OFDM ISAC systems, where both the BS and UT are deployed with Uniform Planar Arrays (UPAs) and hybrid analog–digital beamforming architectures. By combining tensor decomposition with parameter estimation algorithms based on the special properties of matrices, the proposed algorithm achieves excellent ISAC performance in IoT networks. The main contributions of this paper are summarized as follows:

- The proposed algorithm employs tensor decomposition technology for ISAC, i.e., channel parameter estimation and target localization. Firstly, considering the transceiver encoding architecture and the mmWave channel sparse characteristics, the received signal at the UT can be constructed as a third-order tensor model. Moreover, the decomposition of the constructed tensor model is proved to be unique, and an Alternating Least Squares (ALS) scheme can be applied to iteratively estimate the corresponding factor matrices.
- Based on estimated factor matrices, we design specific parameter estimation algorithms to achieve ISAC. Firstly, the ToA expression is derived by utilizing the maximum likelihood principle and the distribution law of the error vector, with the estimated value being obtained through a one-dimensional linear search approach. Then, we use the second-order Taylor expansion to decouple the angle and distance parameters in the near-field channel, approximating the model to a more general form. In addition, we develop a parameter estimation method based on the down sampling covariance matrix, which utilizes the rotational invariance to extract angle parameters.
- The positions of both the Scattering Points (SPs) and UT can be obtained in closed form based on their geometric relationships with the BS, enabling precise localization accuracy. Furthermore, the Cramér–Rao Bounds (CRBs) of channel parameters and positions are derived as theoretical lower bounds. Finally, numerical simulations validate that the proposed tensor-based algorithm achieves superior ISAC performance compared with the existing Phase Unwrapping for Distance Difference (PUDD) [39] and MUSIC-Like Spectrum Peak Searching (MUSIC-LSPS) [40] algorithms, and results are closer to the CRBs.

### 3.2. Notations

We define *x*, x, X and X as scalar, vector, matrix and tensor, respectively. ${\left(\cdot \right)}^{T}$, ${\left(\cdot \right)}^{*}$, ${\left(\cdot \right)}^{H}$, ${\left(\cdot \right)}^{-1}$ and ${\left(\cdot \right)}^{†}$ stand for transpose, conjugate, conjugate transpose, inverse and pseudo-inverse, respectively. diag(x) is the diagonal matrix formed by x. X(m,p) and $X_{:p}$ represent the (m,p)-th element and the *p*-th column of $X\in C^{M\times P}$, respectively. Moreover, ∠(a) denotes the principal angle of the complex scalar *a*. The operator E is indicated as performing mean computations on all vector or matrix components and Re{} refers to the operator that takes the real part of a complex number. The symbols ∘, ⊙, ⊗ and * denote the outer, Khatri–Rao, Kronecker and Hadamard products. ${∥\cdot ∥}_{2}$, ${∥\cdot ∥}_{F}$ and *∂* are the $l_{2}$-norm of the vector, the Frobenius-norm of the matrix and the partial derivative, respectively. Moreover, k(·) expresses the *k*-rank of matrix. $I_{N}$ and $0_{M\times N}$ are the N×N identity and M×N all zero matrix, respectively. Furthermore, we also utilize the property theorem regarding the vectorization of Kronecker products:

(6) $$\mathrm{vec}\left(\mathrm{XYZ}\right)=\left(Z^{T}⊗X\right)\mathrm{vec}\left(Y\right).$$

## 4. System Model

### 4.1. Tensor Representation of Received Signals

**Figure 1:**
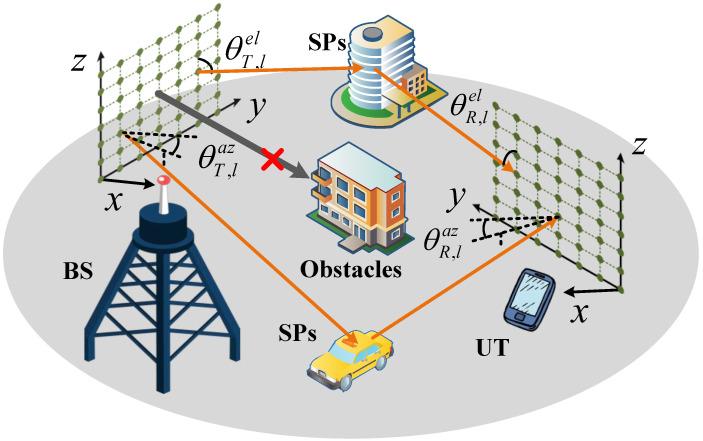
Illustration of the considered near-field mmWave massive MIMO-OFDM ISAC scenario.

**Figure 2:**
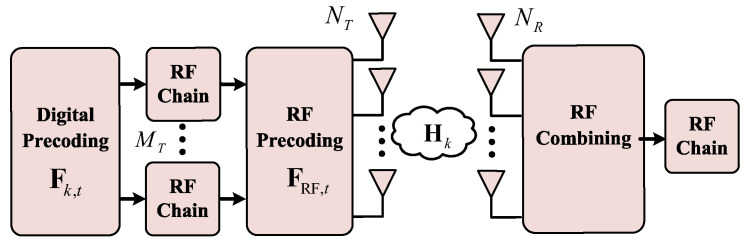
Hybrid analog–digital beamforming architectures of the transmitter and receiver.

As shown in Figure 1, we consider a downlink mmWave massive MIMO-OFDM ISAC system where the BS and UT are equipped with UPAs antennas $N_{T}=N_{T}^{y}\times N_{T}^{z}$ and $N_{R}=N_{R}^{y}\times N_{R}^{z}$ on the yoz plane, with the known location $p_{T}={\left[x_{T},y_{T},z_{T}\right]}^{T}\in R^{3}$ and the unknown location $p_{R}={\left[x_{R},y_{R},z_{R}\right]}^{T}\in R^{3}$, respectively. The *L* SPs located at $p_{l}={\left[x_{l},y_{l},z_{l}\right]}^{T}\in R^{3}$ are randomly distributed within the Fresnel range (i.e., near-field region) between the BS and UT. The range is defined as $D_{T\left(R\right)}=2\left({\left(dN_{T(R)}^{y}\right)}^{2}+{\left(dN_{T(R)}^{z}\right)}^{2}\right)/\lambda$ [41], and each SP satisfies $\left|\right|p_{l}-p_{T(R)}{\left|\right|}_{2}\leq D_{T(R)}$, where d=λ/4 is the antenna spacing, $\lambda =c/f_{c}$ is the wavelength, *c* is the speed of light and $f_{c}$ is the carrier frequency. Additionally, for achieving spatial multiplexing with minimized hardware complexity and power efficiency, hybrid analog–digital beamforming architectures are implemented at both the transmitter and receiver with the detailed architecture depicted in Figure 2. We assume that the BS is equipped with $M_{T}$ Radio-Frequency (RF) chains and the UT is equipped with $M_{R}=1$ RF chains, satisfying $M_{T}<N_{T}$. The important variables are listed in Table 1.

Considering the anti-interference ability of OFDM technology in frequency-selective fading channels, it is applied in this system to improve the spectral efficiency. Similar to [42], the training scheme based on OFDM is used to construct the received signal model. The BS transmits data to the UT over $\bar{K}$ subcarriers, with *K* subcarriers selected for pilot training. Assume that each subcarrier consists of *T* consecutive time frames corresponding to *T* distinct beamforming vectors, and each frame is composed of *F* sub-frames. Let $f_{k,t}\in C^{M_{T}\times 1}$ denote the beamforming vector for subcarrier *k* at time frame *t*, given by

(7) $$f_{k,t}=F_{\mathrm{RF},t}F_{k,t}s_{k,t},$$

where $F_{\mathrm{RF},t}\in C^{N_{T}\times M_{T}}$ is a RF precoding matrix that is the same for all subcarriers, $F_{k,t}\in C^{M_{T}\times S}$ expresses the digital precoding matrix for the subcarrier *k*, and $s_{k,t}\in C^{S\times 1}$ is a column vector with *S* pilot symbols. The receiving end processes the transmitted signal in units of sub-frames. Thus, at each time frame, the UT successively employs *F* RF combining vectors $w_{f}\in C^{N_{R}\times 1}$ to detect the received signal. Note that the same combining vectors are applied uniformly to every subcarrier in the training scheme. In order to construct the received signal as a tensor model with low-rank characteristics, we suppose the digital precoding matrices $F_{k,t}$ and pilot symbols vector $s_{k,t}$ are invariant with respect to subcarrier index, i.e., $F_{k,t}=F_{t}$, $s_{k,t}=s_{t}$ and $f_{k,t}=f_{t}$. The received signal for the *k*-th subcarrier in the *f*-th sub-frame within the *t*-th time frame can be formulated as

(8) $$y_{k,f,t}=w_{f}^{T}H_{k}f_{t}+v_{k,f,t},$$

where $H_{k}\in C^{N_{R}\times N_{T}}$ is the near-field channel matrix and $v_{k,f,t}$ is the additive Gaussian noise, respectively. Gathering the received signals on *F* sub-frames and arranging them into column vector can yield the following expression

(9) $$y_{k,t}=W^{T}H_{k}f_{t}+v_{k,t}\in C^{F\times 1},$$

where $W=\left[w_{1},w_{2},\ldots ,w_{F}\right]\in C^{N_{R}\times F}$ and $v_{k,t}={\left[v_{k,1,t},v_{k,2,t},\ldots ,v_{k,F,t}\right]}^{T}\in C^{F\times 1}$. Similarly, the precoding vector $f_{t}$ is extended to a precoding matrix $F=\left[f_{1},f_{2},\ldots ,f_{T}\right]\in C^{N_{T}\times T}$, with each column corresponding to a time frame. Thus, the received signal at UT can be represented in matrix form, i.e.,

(10) $$Y_{k}=W^{T}H_{k}F+V_{k}\in C^{F\times T},$$

where $V_{k}=\left[v_{k,1},v_{k,2},\ldots ,v_{k,T}\right]$ is the noise matrix.

The mmWave suffers from significant path loss because of its limited propagation range and weak penetration ability, making the Line-of-Sight (LoS) path particularly vulnerable to obstruction [43]. Therefore, only the Non-LoS (NLoS) path is considered in this paper. Since the mmWave propagate mainly in straight lines with minimal diffraction, they have fewer scattering paths. This sparse multipath nature allows for clear geometric channel properties, which can be leveraged for precision channel modeling [44]. Therefore, the mmWave time-domain channel impulse response can be expressed as

(11) $$H\left(\tau \right)=\sum_{l=1}^{L}\alpha_{l}a_{R,l}\left(\theta_{R,l}^{az},\theta_{R,l}^{el},d_{R,l}^{c}\right)b_{T,l}^{T}\left(\theta_{T,l}^{az},\theta_{T,l}^{el},d_{T,l}^{c}\right)\delta \left(\tau -\tau_{l}\right),$$

where δ(·) represents the delta function, $\tau_{l}$ is the ToA for path *l*, $d_{R,l}^{c}$ and $d_{T,l}^{c}$ represent the distances from the *l*-th SP to the centers of receiver and transmitter arrays, $\alpha_{l}$ is the complex gain for path *l*, and $\theta_{R(T),l}^{az}$ and $\theta_{R(T),l}^{el}$ are the azimuth and elevation angles at the UT (BS), respectively. The $n_{R}$-th and $n_{T}$-th elements of antenna array response vectors $a_{R,l}\in C^{N_{R}\times 1}$ and $b_{T,l}\in C^{N_{T}\times 1}$ at the UT and BS are represented, respectively, as [45]

(12) $$a_{R,l}\left(n_{R}\right)=e^{-j\frac{2\pi }{\lambda }\left(d_{R,l}^{n_{R}}-d_{R,l}^{c}\right)},b_{T,l}\left(n_{T}\right)=e^{-j\frac{2\pi }{\lambda }\left(d_{T,l}^{n_{T}}-d_{T,l}^{c}\right)},$$

where $n_{R(T)}=\left(N_{R(T)}^{y}N_{R(T)}^{z}+1\right)/2+n_{R(T)}^{z}N_{R(T)}^{y}+n_{R(T)}^{y}$. The antenna indexes $n_{R\left(T\right)}^{y}$ and $n_{R\left(T\right)}^{z}$ along *y* and *z* directions belong to $\left\{-\left(N_{R\left(T\right)}^{y}-1\right)/2,\ldots ,0,\ldots ,\left(N_{R\left(T\right)}^{y}-1\right)/2\right\}$ and $\left\{-\left(N_{R\left(T\right)}^{z}-1\right)/2,\ldots ,0,\ldots ,\left(N_{R\left(T\right)}^{z}-1\right)/2\right\}$, respectively. To solve the delay spread problem caused by the multipath effect in the wideband mmWave system, the ToA $\tau_{l}$ is represented as the phase shift on the subcarrier through Fourier transform for better analysis and parameter extraction. Specifically, we have

(13) $$H\left(f\right)=\sum_{l=1}^{L}\alpha_{l}a_{R,l}b_{T,l}^{T}\int_{-\infty }^{\infty }\delta \left(\tau -\tau_{l}\right)e^{-j2\pi f\tau }d\tau =\sum_{l=1}^{L}\alpha_{l}e^{-j2\pi f\tau_{l}}a_{R,l}b_{T,l}^{T}.$$

Furthermore, the frequency-domain channel at the *k*-th subcarrier can be expressed as

(14) $$H_{k}=\sum_{l=1}^{L}\alpha_{l}e^{-j2\pi \tau_{l}f_{s}k/\bar{K}}a_{R,l}b_{T,l}^{T},$$

where $f_{s}$ is the sampling frequency.

Substituting (14) into (10) yields the result

(15) $$Y_{k}=\sum_{l=1}^{L}\alpha_{l}e^{-j2\pi \tau_{l}f_{s}k/\bar{K}}W^{T}a_{R,l}b_{T,l}^{T}F+V_{k}.$$

Since the signal associated with multiple subcarriers is available, the received signal ${\left\{Y_{k}\right\}}_{k=1,\ldots ,K}$ can be constructed as a third-order tensor $Y\in C^{F\times T\times K}$ whose three dimensions represent the sub-frames, the time frames and the subcarriers, respectively. Each slice of the tensor Y, i.e., $Y_{k}$, is a weighted sum of rank-one outer products, which gives the received signal tensor Y a low-rank structure. This property ensures the uniqueness of its tensor decomposition. Therefore, the received signal follows CP decomposition which decomposes tensor as

(16) Y=∑l=1L(WTaR,l)∘FTbT,l∘cl+V=I3,L×WTAR1×FTBT2×C3+V,

where $c_{l}=\alpha_{l}\bar{c}\left(\tau_{l}\right)=\alpha_{l}{\left[e^{-j2\pi \tau_{l}f_{s}/\bar{K}},e^{-j2\pi \tau_{l}f_{s}2/\bar{K}},\ldots ,e^{-j2\pi \tau_{l}f_{s}K/\bar{K}}\right]}^{T}\in C^{K\times 1}$, $A_{R}=\left[a_{R,1},\ldots ,a_{R,L}\right]\in C^{N_{R}\times L}$, $B_{T}=\left[a_{T,1},\ldots ,a_{T,L}\right]\in C^{N_{T}\times L}$, $C=\left[c_{1},\ldots ,c_{L}\right]\in C^{K\times L}$, $I_{3,L}$ is the identity tensor with size L×L×L and $V\in C^{F\times T\times K}$ is a third-order noise tensor.

### 4.2. Second-Order Taylor Expansion

Different from the far-field communication, there is a nonlinear relationship between the angle and distance parameters for the near-field spherical wave model. Thus, we use the second-order Taylor expansion to approximate the model, enabling a transition from the near-field spherical wave model to the far-field plane wave model. Next, we take $a_{R,l}$ as an example to discuss the approximation process. Firstly, exploiting the geometric relationship between the UT and SP, $d_{R,l}^{n_{R}}$ can be given by

(17) $$\begin{array}{cc} d_{R,l}^{n_{R}} & ={\left({\left(d_{R,l}^{c}\right)}^{2}+{\left(n_{R}^{z}d\right)}^{2}+{\left(n_{R}^{y}d\right)}^{2}-2n_{R}^{y}dd_{R,l}^{c}\sin\theta_{R,l}^{az}\sin\theta_{R,l}^{el}+2n_{R}^{z}dd_{R,l}^{c}\cos\theta_{R,l}^{el}\right)}^{\frac{1}{2}}\\ \; & =d_{R,l}^{c}{\left(1+z^{2}+y^{2}-2y\sin\theta_{R,l}^{az}\sin\theta_{R,l}^{el}+2z\cos\theta_{R,l}^{el}\right)}^{\frac{1}{2}}\\ \; & =d_{R,l}^{c}\Gamma \left(y,z\right), \end{array}$$

where $y=n_{R}^{y}d/d_{R,l}^{c}$ and $z=n_{R}^{z}d/d_{R,l}^{c}$. By performing the second-order Taylor expansion on Γ(y,z), we then have the approximate form as follows:

(18) $$\Gamma \left(y,z\right)\approx 1-\beta_{R,l}y+\sigma_{R,l}z+\frac{1}{2}\left({\bar{\beta }}_{R,l}y^{2}+{\bar{\sigma }}_{R,l}z^{2}+2\sigma_{R,l}\beta_{R,l}yz\right),$$

where $\sigma_{R,l}=\cos\theta_{R,l}^{el}$, $\beta_{R,l}=\sin\theta_{R,l}^{az}\sin\theta_{R,l}^{el}$, ${\bar{\sigma }}_{R,l}=1-\sigma_{R,l}^{2}$ and ${\bar{\beta }}_{R,l}=1-\beta_{R,l}^{2}$. Thus, $d_{R,l}^{n_{R}}$ can be simplified as

(19) $$\begin{array}{cc} d_{R,l}^{n_{R}} & \approx \underset{\Phi_{n_{R}}\left(\sigma_{R,l},\beta_{R,l},d_{R,l}^{c}\right)}{\underset{︸}{-\frac{{\left(n_{R}^{y}d\beta_{R,l}-n_{R}^{z}d\sigma_{R,l}\right)}^{2}}{2d_{R,l}^{c}}+\frac{1}{2d_{R,l}^{c}}\left({\left(n_{R}^{z}d\right)}^{2}+{\left(n_{R}^{y}d\right)}^{2}\right)}}+\underset{\Xi_{n_{R}}\left(\sigma_{R,l},\beta_{R,l}\right)}{\underset{︸}{n_{R}^{z}d\sigma_{R,l}-n_{R}^{y}d\beta_{R,l}}}+d_{R,l}^{c}. \end{array}$$

Based on the above derivation, $a_{R,l}$ can be reformulated as

(20) $$\begin{array}{c} a_{R,l}\left(n_{R}\right)\approx e^{-j\frac{2\pi }{\lambda }\left(\Xi_{n_{R}}+\Phi_{n_{R}}\right)}, \end{array}$$

whose covariance matrix $R=\sum_{l=1}^{L}a_{R,l}a_{R,l}^{H}$ satisfies

(21) $$R\left(n_{R},{\tilde{n}}_{R}\right)\approx \sum_{l=1}^{L}e^{-j\frac{2\pi }{\lambda }\left(\Xi_{n_{R},{\tilde{n}}_{R}}+\Phi_{n_{R},{\tilde{n}}_{R}}\right)},$$

where $\Xi_{n_{R},{\tilde{n}}_{R}}=\Xi_{n_{R}}-\Xi_{{\tilde{n}}_{R}}$, $\Phi_{n_{R},{\tilde{n}}_{R}}=\Phi_{n_{R}}-\Phi_{{\tilde{n}}_{R}}$, and ${\tilde{n}}_{R}=\left(N_{R}^{y}N_{R}^{z}+1\right)/2+{\tilde{n}}_{R}^{z}N_{R}^{y}+{\tilde{n}}_{R}^{y}$. When $n_{R}^{y}=-{\tilde{n}}_{R}^{y}$ and $n_{R}^{z}=-{\tilde{n}}_{R}^{z}$, we obtain $\Xi_{n_{R},{\tilde{n}}_{R}}=2d\sigma_{R,l}n_{R}^{z}-2d\beta_{R,l}n_{R}^{y}$ and $\Phi_{n_{R},{\tilde{n}}_{R}}=0$. Then, the (21) can be simplified as

(22) $$R\left(n_{R},{\tilde{n}}_{R}\right)\approx \sum_{l=1}^{L}e^{-j\frac{2\pi }{\lambda }\Xi_{n_{R},{\tilde{n}}_{R}}}.$$

Based on the above, we realize the decoupling of angle and distance parameters. Then, the general far-field array response can be defined as

(23) $${\tilde{a}}_{R,l}\left(v_{R}\right)=e^{-j\frac{2\pi }{\lambda }\left(2d\sigma_{R,l}v_{R}^{z}-2d\beta_{R,l}v_{R}^{y}\right)},$$

where $v_{R}=v_{R}^{z}\left(N_{R}^{y}+1\right)/2+v_{R}^{y}+1$ and $v_{R}^{y(z)}=0,\ldots ,\left(N_{R}^{y(z)}-1\right)/2$. The covariance matrix of ${\tilde{a}}_{R,l}$ is expressed as

(24) $$\tilde{R}\left(v_{R},{\tilde{v}}_{R}\right)=\sum_{l=1}^{L}e^{-j\frac{2\pi }{\lambda }\left(2d\sigma_{R,l}\left(v_{R}^{z}-{\tilde{v}}_{R}^{z}\right)-2d\beta_{R,l}\left(v_{R}^{y}-{\tilde{v}}_{R}^{y}\right)\right)},$$

where ${\tilde{v}}_{R}={\tilde{v}}_{R}^{z}\left(N_{R}^{y}+1\right)/2+{\tilde{v}}_{R}^{y}+1$. From the (22) and (24), it can be observed that when $n_{R}=\left(N_{R}^{y}N_{R}^{z}+1\right)/2+\left(v_{R}^{z}-{\tilde{v}}_{R}^{z}\right)N_{R}^{y}+\left(v_{R}^{y}-{\tilde{v}}_{R}^{y}\right)$ and ${\tilde{n}}_{R}=\left(N_{R}^{y}N_{R}^{z}+1\right)/2-\left(v_{R}^{z}-{\tilde{v}}_{R}^{z}\right)N_{R}^{y}-\left(v_{R}^{y}-{\tilde{v}}_{R}^{y}\right)$ hold true, $R\left(n_{R},{\tilde{n}}_{R}\right)=\tilde{R}\left(v_{R},{\tilde{v}}_{R}\right)$ can be obtained. By applying the second-order Taylor expansion, the constructed near-field model can be approximated to a simpler form. Note that this approximation process is only a theoretical approximation. In an actual simulation, some signals are extracted and processed as plane wave models, which will be introduced in the next section.

## 5. The Proposed Tensor-Based Algorithm

### 5.1. CP Decomposition

The Minimum Description Length (MDL) [46] is an effective approach for achieving the rank estimation of multilinear tensors. Since the rank of the mode expansion of tensor Y corresponds to the number of paths, the MDL method can be applied to estimate *L*. The mode-1, mode-2 and mode-3 unfoldings of tensor Y are, respectively, denoted as

(25) $$\begin{array}{cc} {\left[Y\right]}_{\left(1\right)}^{T} & =\hat{A}{\left(\hat{C}⊙\hat{B}\right)}^{T}+{\left[V\right]}_{\left(1\right)}^{T},\\ {Y]}_{\left(2\right)}^{T} & =\hat{B}{\left(\hat{C}⊙\hat{A}\right)}^{T}+{\left[V\right]}_{\left(2\right)}^{T},\\ {Y]}_{\left(3\right)}^{T} & =\hat{C}{\left(\hat{B}⊙\hat{A}\right)}^{T}+{\left[V\right]}_{\left(3\right)}^{T}, \end{array}$$

where ${\left[V\right]}_{\left(1\right)}^{T}$, ${\left[V\right]}_{\left(2\right)}^{T}$ and ${\left[V\right]}_{\left(3\right)}^{T}$ are the mode-1, mode-2 and mode-3 unfoldings of V. To simplify the expression, the mode-*n* unfolding of tensor Y is defined as ${\left[Y\right]}_{\left(n\right)}^{T}$ and *F*, *T* and *K* are replaced by $J_{1}$, $J_{2}$ and $J_{3}$, where n∈{1,2,3}. By performing eigenvalue decomposition on the sample covariance matrix ${\left[Y\right]}_{\left(n\right)}^{T}{\left[Y\right]}_{\left(n\right)}^{*}J_{n}/J_{1}J_{2}J_{3}$, the eigenvalues $\left\{\varepsilon_{1}^{n},\ldots ,\varepsilon_{J_{n}}^{n}\right\}$ can be obtained. The estimated value of *L* is $\hat{L}=\min{\left\{{\hat{L}}_{n}\right\}}_{n=1}^{3}$, where

(26) $${\hat{L}}_{n}=\arg\min_{l_{n}\in \left\{0,1,\ldots ,J_{n}-1\right\}}\mathrm{MDL}\left(l_{n}\right),$$

and

(27) $$\mathrm{MDL}\left(l_{n}\right)=-2J_{1}J_{2}J_{3}/J_{n}\left(J_{n}-l_{n}\right)\log\left(\frac{\prod_{\zeta =l_{n}+1}^{J_{n}}{\left(\varepsilon_{\zeta }^{\left(n\right)}\right)}^{\frac{1}{J_{n}-l_{n}}}}{\frac{1}{J_{n}-l_{n}}\sum_{\zeta =l_{n}+1}^{J_{n}}\varepsilon_{\zeta }^{\left(n\right)}}\right)+l_{n}\left(2J_{n}-l_{n}\right)\log\left(J_{1}J_{2}J_{3}/J_{n}\right).$$

According to (16), we define factor matrices $A=W^{T}A_{R}\in C^{F\times L}$ and $B=F^{T}B_{T}\in C^{T\times L}$. For the convenience of subsequent description, we set the column vectors corresponding to matrices A and B as $a_{l}$ and $b_{l}$, respectively. The CP decomposition of tensor Y can be accomplished by solving

(28) $$\min_{\hat{A},\hat{B},\hat{C}}{\left\|Y-\sum_{l=1}^{L}{\hat{a}}_{l}\circ {\hat{b}}_{l}\circ {\hat{c}}_{l}\right\|}_{F}^{2}.$$

The fitting error can be effectively minimized through an ALS method that iteratively optimizes each factor matrix while holding the remaining two factor matrices constant. Firstly, the initial values for the iteration, ${\hat{A}}_{0}$, ${\hat{B}}_{0}$ and ${\hat{C}}_{0}$, are formed by the first *L* left singular vectors of ${\left[Y\right]}_{\left(1\right)}$, ${\left[Y\right]}_{\left(2\right)}$ and ${\left[Y\right]}_{\left(3\right)}$, respectively. Then, the factor matrices are updated iteratively through the following formulas

(29) $$\begin{array}{cc} {\hat{A}}_{m+1} & =\underset{\hat{A}}{\arg\min}{\left\|{\left[Y\right]}_{\left(1\right)}-\left({\hat{C}}_{m}⊙{\hat{B}}_{m}\right){\hat{A}}^{T}\right\|}_{F}^{2},\\ {\hat{B}}_{m+1} & =\underset{\hat{B}}{\arg\min}{\left\|{\left[Y\right]}_{\left(2\right)}-\left({\hat{C}}_{m}⊙{\hat{A}}_{m+1}\right){\hat{B}}^{T}\right\|}_{F}^{2},\\ {\hat{C}}_{m+1} & =\underset{\hat{C}}{\arg\min}{\left\|{\left[Y\right]}_{\left(3\right)}-\left({\hat{B}}_{m+1}⊙{\hat{A}}_{m+1}\right){\hat{C}}^{T}\right\|}_{F}^{2}, \end{array}$$

whose least squares solution can be expressed as

(30) $$\begin{array}{cc} {\hat{A}}_{m+1} & ={\left[Y\right]}_{\left(1\right)}\left({\hat{C}}_{m}⊙{\hat{B}}_{m}\right){\left[({\hat{C}}_{m}^{T}{\hat{C}}_{m}*{\hat{B}}_{m}^{T}{\hat{B}}_{m})\right]}^{-1},\\ {\hat{B}}_{m+1} & ={\left[Y\right]}_{\left(2\right)}\left({\hat{C}}_{m}⊙{\hat{A}}_{m+1}\right){\left[({\hat{C}}_{m}^{T}{\hat{C}}_{m}*{\hat{A}}_{m+1}^{T}{\hat{A}}_{m+1})\right]}^{-1},\\ {\hat{C}}_{m+1} & ={\left[Y\right]}_{\left(3\right)}\left({\hat{B}}_{m+1}⊙{\hat{A}}_{m+1}\right){\left[({\hat{B}}_{m+1}^{T}{\hat{B}}_{m+1}*{\hat{A}}_{m+1}^{T}{\hat{A}}_{m+1})\right]}^{-1}, \end{array}$$

where we set $\left\{\hat{A},\hat{B},\hat{C}\right\}$ as the subspace spanned by $\left\{A,B,C\right\}$ and *m* expresses the *m*-th iteration, respectively. The (30) is applied to update the factor matrix alternately until the convergence condition $\|Y-\sum_{l=1}^{L}{\hat{a}}_{l}\circ {\hat{b}}_{l}\circ {\hat{c}}_{l}{\|}_{F}^{2}$ is met for a certain error threshold ε>0, then the iteration stops, getting the estimated factor matrices $\left\{\hat{A},\hat{B},\hat{C}\right\}$. It is worth noting that in actual communication scenarios, the received signals need to be represented with higher dimensions, and the ALS method can still be used for the decomposition of high-order tensor models. For instance, in reference [47], a stable ALS method was developed for iteratively fitting a sixth-order tensor model for real-time communication systems. In addition, work [48] proposed a five-linear ALS to achieve inner tensor decomposition for the MIMO ISAC systems.

### 5.2. Parameter Estimation

To preserve the internal structures of matrices $A_{R}$ and $B_{T}$ and ensure the parameter extraction based on rotational invariance remains effective, matrices W and F are designed as truncated Discrete Fourier Transform (DFT) matrices. Leveraging the column orthogonality of W and F, i.e., $WW^{T}=I_{N_{R}}$ and $FF^{T}=I_{N_{T}}$, $A_{R}$ and $B_{T}$ can be estimated by

(31) $${\hat{A}}_{R}={\left(W^{T}\right)}^{†}\hat{A},{\hat{B}}_{T}={\left(F^{T}\right)}^{†}\hat{B}.$$

According to estimated factor matrices $\hat{C}$ and the distribution characteristics of error vector, the ToA can be obtained via column correlation

(32) $$\hat{\tau_{l}}=arg\max_{\tau_{l}}\frac{\left|{\hat{c}}_{l}^{H}\bar{c}\left(\tau_{l}\right)\right|}{{∥\bar{c}\left(\tau_{l}\right)∥}_{2}{∥{\hat{c}}_{l}∥}_{2}},$$

where ${\hat{c}}_{l}$ is the *l*-th column of $\hat{C}$. Specifically, we employ a one-dimensional exhaustive search method with $N_{s}$ uniformly distributed sampling points to traverse the entire sampling set. The detailed derivation of the (32) can be found in Appendix A.

For conciseness, we illustrate the angle extraction process using AoAs estimation at the UT. According to Section 3, the approximate covariance matrix $\tilde{R}$ can be obtained by performing down sampling for $\hat{R}={\hat{A}}_{R}{\hat{A}}_{R}^{H}$. Based on $\hat{R}\left(n_{R},{\tilde{n}}_{R}\right)=\tilde{R}\left(v_{R},{\tilde{v}}_{R}\right)$, we can extract some elements from $\hat{R}$ and rearrange them as

(33) $$\tilde{R}=\left[\begin{array}{ccc} {\tilde{R}}_{0} & \ldots & {\tilde{R}}_{-W_{y}}\\ \vdots & \ddots & \vdots \\ {\tilde{R}}_{W_{y}} & \ldots & {\tilde{R}}_{0} \end{array}\right],$$

where

(34) $${\tilde{R}}_{n_{R}^{y}}=\left[\begin{array}{ccc} e\left(n_{R}^{y},0\right) & \ldots & e\left(n_{R}^{y},-W_{z}\right)\\ \vdots & \ddots & \vdots \\ e\left(n_{R}^{y},W_{z}\right) & \ldots & e\left(n_{R}^{y},0\right) \end{array}\right],$$

where $W_{y}=\frac{N_{R}^{y}-1}{2}$, $W_{z}=\frac{N_{R}^{z}-1}{2}$ and $e\left(n_{R}^{y},n_{R}^{z}\right)=\hat{R}\left(n_{R}\left(n_{R}^{y},n_{R}^{z}\right),{\tilde{n}}_{R}\left(-n_{R}^{y},-n_{R}^{z}\right)\right)$ is a function about $n_{R}^{y}$ and $n_{R}^{z}$. Considering the general structure of far-field response vectors, we develop a rotational invariance-based algorithm to estimate angle parameters. Firstly, the singular value decomposition of the covariance matrix $\tilde{R}$ is given by

(35) $$\tilde{R}=U\Sigma G^{H},$$

where Σ expresses a diagonal matrix consisting of the singular values and G and U are the right and left singular matrices, respectively. The first *L* column vectors of U constitutes the matrix $U_{:L}$ including the angle parameter information to be estimated. The parameter extraction process of $U_{:L}$ is as follows:

(36) $$U_{1}\Psi_{R}^{y}=U_{2},U_{3}\Psi_{R}^{z}=U_{4},$$

where $\Psi_{R}^{y}\in C^{L\times L}$ and $\Psi_{R}^{z}\in C^{L\times L}$ are the rotation factors corresponding to the UT along the *y* and *z* directions, respectively. The four sub-matrices of $U_{:L}$ are expressed as

(37) $$U_{1(2)}=\left(I_{W_{z}}⊗J_{1(2)}\right)U_{:L},U_{3(4)}=\left(J_{3(4)}⊗I_{W_{y}}\right)U_{:L},$$

where $J_{1}$, $J_{2}$, $J_{3}$ and $J_{4}$ are selection matrices, i.e.,

(38) $$J_{1(3)}=\left[I_{W_{y(z)}},0_{W_{y(z)}\times 1}\right],J_{2(4)}=\left[0_{W_{y(z)}\times 1},I_{W_{y(z)}}\right].$$

Thus, the rotation factors $\Psi_{R}^{y}$ and $\Psi_{R}^{z}$ are obtained by

(39) $$\begin{array}{cc} {\hat{\Psi }}_{R}^{y}= & {\left(\left(I_{W_{z}}⊗J_{1}\right)U_{:L}\right)}^{†}\left(\left(I_{W_{z}}⊗J_{2}\right)U_{:L}\right),\\ {\hat{\Psi }}_{R}^{z}= & {\left(\left(J_{3}⊗I_{W_{y}}\right)U_{:L}\right)}^{†}\left(\left(J_{4}⊗I_{W_{y}}\right)U_{:L}\right). \end{array}$$

Therefore, we can estimate the AoAs $\left\{{\hat{\theta }}_{R,l}^{az},{\hat{\theta }}_{R,l}^{el}\right\}$ from the eigenvalues ${\left[\lambda_{R,l}^{y(z)}\right]}_{l=1}^{L}$ of $\Psi_{R}^{y(z)}$ as

(40) $$\begin{array}{cc} {\hat{\theta }}_{R,l}^{el} & =\arccos(-∠\left(\lambda_{R,l}^{z}\right)/\pi ),\\ {\hat{\theta }}_{R,l}^{az} & =\arcsin(-∠\left(\lambda_{R,l}^{y}\right)/\pi /\sin\left({\hat{\theta }}_{R,l}^{el}\right)). \end{array}$$

Similarly, the AoDs $\left\{{\hat{\theta }}_{T,l}^{az},{\hat{\theta }}_{T,l}^{el}\right\}$ can be obtained from the eigenvalues ${\left[\lambda_{T,l}^{y(z)}\right]}_{l=1}^{L}$ of $\Psi_{T}^{y(z)}$.

### 5.3. UT and SPs Localization

In this subsection, we develop a spatial information-based three-dimensional (3D) localization algorithm to simultaneously determine UT and SPs positions through statistical likelihood maximization, i.e.,

(41) $$\left[{\hat{p}}_{R},{\left[{\hat{p}}_{l}\right]}_{l=1}^{L}\right]=\underset{p_{R},{\left[p_{l}\right]}_{l=1}^{L}}{\arg\max}p\left({\left[\hat{\tau_{l}},{\hat{\theta }}_{R,l}^{az},{\hat{\theta }}_{R,l}^{el},{\hat{\theta }}_{T,l}^{az},{\hat{\theta }}_{T,l}^{el}\right]}_{l=1}^{L}|p_{R},{\left[p_{l}\right]}_{l=1}^{L},p_{T}\right).$$

The vector $g_{T,l}={\left[\cos{\hat{\theta }}_{T,l}^{az}\sin{\hat{\theta }}_{T,l}^{el},\sin{\hat{\theta }}_{T,l}^{az}\sin{\hat{\theta }}_{T,l}^{el},\cos{\hat{\theta }}_{T,l}^{el}\right]}^{T}$ is defined as the transmitting direction along the *l*-th path. Likewise, $g_{R,l}\in R^{3}$ is expressed as the receiving direction along the *l*-th path. The geometric relationship between the UT, SPs and BS can be described as

(42) $$\begin{array}{cc} {\hat{p}}_{R} & =p_{T}+c\hat{\tau_{l}}\upsilon_{l}g_{T,l}-c\hat{\tau_{l}}\left(1-\upsilon_{l}\right)g_{R,l}\\ \; & =\eta_{l}+\upsilon_{l}u_{l}, \end{array}$$

where $\upsilon_{l}\in \left[0,1\right]$ represents the time delay ratio from the BS to the *l*-th SP, $\eta_{l}=p_{T}-c\hat{\tau_{l}}g_{R,l}$ and $u_{l}=c\hat{\tau_{l}}\left(g_{T,l}+g_{R,l}\right)$. Due to $\upsilon_{l}{∥u∥}^{2}=u_{l}^{T}\left(\upsilon_{l}u_{l}\right)=u_{l}^{T}\left(p_{R}-\eta_{l}\right)$, ${\hat{p}}_{R}$ can be rewritten as

(43) $${\hat{p}}_{R}=\eta_{l}+{\bar{u}}_{l}^{T}\left(p_{R}-\eta_{l}\right){\bar{u}}_{l},$$

where ${\bar{u}}_{l}=u_{l}/∥u_{l}∥$. Thus, the position $p_{R}$ of UT can be gained by minimizing the following loss function

(44) $$L\left(p_{R}\right)=\sum_{l=1}^{L}\xi_{l}{∥p_{R}-\left(\eta_{l}+{\bar{u}}_{l}^{T}\left(p_{R}-\eta_{l}\right){\bar{u}}_{l}\right)∥}_{2}^{2},$$

where $\xi_{l}\geq 0$ is the weight of path *l*. Furthermore, the position of UT can be obtained as

(45) $${\hat{p}}_{R}={\left(\sum_{l=1}^{L}\xi_{l}\left(I_{3}-{\bar{u}}_{l}{\bar{u}}_{l}^{T}\right)\right)}^{-1}\sum_{l=1}^{L}\xi_{l}\left(I_{3}-{\bar{u}}_{l}{\bar{u}}_{l}^{T}\right)\eta_{l}.$$

Since the SPs lie at the intersections of the direction vectors $g_{T,l}$ and $g_{R,l}$, their distances to the BS and UT can be expressed as

(46) $$\begin{array}{cc} d_{T,l}^{c} & =g_{T,l}^{T}\left(p_{l}-p_{T}\right)/{∥g_{T,l}∥}^{2},\\ d_{R,l}^{c} & =g_{R,l}^{T}\left(p_{l}-{\hat{p}}_{R}\right)/{∥g_{R,l}∥}^{2}. \end{array}$$

**Figure 3:**
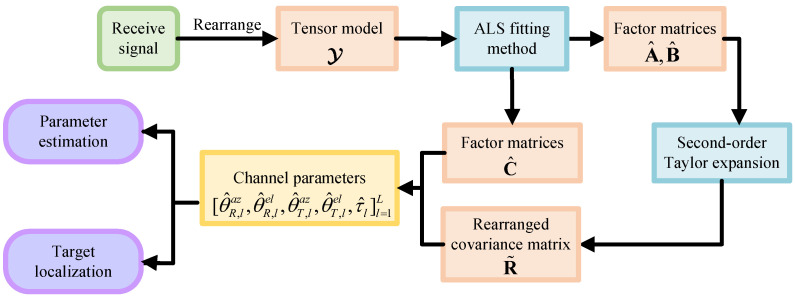
Block diagram of tensor-based ISAC algorithm in near-field mmWave massive MIMO-OFDM systems.

Substituting the (46) into $p_{T}+d_{T,l}^{c}g_{T,l}+{\hat{p}}_{R}+d_{R,l}^{c}g_{R,l}=2p_{l}$, the position $p_{l}$ can be computed by

(47) $${\hat{p}}_{l}={\left(Q_{T,l}+Q_{R,l}\right)}^{-1}\left(Q_{T,l}p_{T}+Q_{R,l}{\hat{p}}_{R}\right),$$

where $Q_{T,l}=I_{3}-g_{T,l}g_{T,l}^{T}$ and $Q_{R,l}=I_{3}-g_{R,l}g_{R,l}^{T}$. Finally, the proposed tensor-based channel parameter estimation and localization algorithm is shown in Algorithm 1. In addition, the algorithm process is presented in the form of a block diagram in Figure 3.

**Algorithm 1:** The proposed tensor-based channel parameter estimation and localization algorithm
**Input:** The combining matrix W, precoding matrix F, received tensor Y, error threshold $\varepsilon =1\times 10^{-10}$, number of iterations *M* and sampling points $N_{s}$
1: Take the first *L* left singular vectors of ${\left[Y\right]}_{\left(1\right)}$, ${\left[Y\right]}_{\left(2\right)}$ and ${\left[Y\right]}_{\left(3\right)}$ as the initial factor matrices ${\hat{A}}_{0}$, ${\hat{B}}_{0}$ and ${\hat{C}}_{0}$
2. Set m=0
3: **While** ${\left|\right|{\hat{Y}}_{m}-{\hat{Y}}_{m+1}\left|\right|}_{2}/{\left|\right|{\hat{Y}}_{m}\left|\right|}_{2}>\varepsilon$ or m<M
      (a) Update factor matrices $\hat{A}$, $\hat{B}$, $\hat{C}$ by (29)
      (b) Reconstruct ${\hat{Y}}_{m}$
      *m* = *m* + 1
   **End while**
4: Get ${\hat{A}}_{R}$ and ${\hat{B}}_{T}$ by (31)
5: Compute ${\left[{\hat{\tau }}_{1}\right]}_{l=1}^{L}$ by (32)
6: Get down sampling of covariance matrices corresponding to ${\hat{A}}_{R}$ and ${\hat{B}}_{T}$
7: Obtain ${\left[{\hat{\theta }}_{R,l}^{az},{\hat{\theta }}_{R,l}^{el},{\hat{\theta }}_{T,l}^{az},{\hat{\theta }}_{T,l}^{az}\right]}_{l=1}^{L}$ by (33)–(40)
8: Get ${\hat{p}}_{R}$ and ${\left[{\hat{p}}_{l}\right]}_{l=1}^{L}$ by (45) and (47)
**Output:**${\left[{\hat{\theta }}_{R,l}^{az},{\hat{\theta }}_{R,l}^{el},{\hat{\theta }}_{T,l}^{az},{\hat{\theta }}_{T,l}^{el},{\hat{\tau }}_{l},{\hat{p}}_{l}\right]}_{l=1}^{L}$, ${\hat{p}}_{R}$


## 6. Uniqueness and Complexity

### 6.1. Uniqueness

For tensor Y composed of *L* rank-1 matrices, according to Kruskal’s condition, if

(48) k(A)+k(B)+k(C)≥2L+2,

then estimated factor matrices $\hat{A}$, $\hat{B}$, $\hat{C}$ are unique. Although the condition (48) is not satisfied when L=1, the uniqueness of decomposition in this case has been proved by Harshman in [49]. Obviously, Kruskal’s condition is sufficient and necessary in the case of L=2. The decomposition uniqueness when L≥3 needs to be further discussed based on the model.

Note that the column vectors of $A_{R}=\left[a_{R,1},\ldots ,a_{R,L}\right]$ are linearly independent when the assumption that the SPs are at distinct positions holds true. Since $W^{T}$ is the column orthogonal DFT matrix, the column space linear dependence structure of matrix $A_{R}$ is invariant under left multiplication by matrix $W^{T}$. Therefore, k(A)=min(F,L) can be inferred. Similarly, it can be obtained that k(B)=min(T,L). Since that matrix C is a Vandermonde matrix scaled by column and $\tau_{l_{1}}\neq \tau_{l_{2}}$, it follows full column rank structure when K≥L. Considering the number of scatterers *L* is small in the real near-field, it is reasonable to assume F≥L and T≥L for avoiding reducing the ranks of matrices $A_{R}$ and $B_{T}$. Therefore, A, B and C are full column rank, i.e., k(A)=L, k(B)=L, k(C)=L, which means that the uniqueness condition is valid at L≥2.

### 6.2. Complexity

The computational complexity of the proposed tensor-based channel parameter estimation and localization algorithm mainly consists of two components, i.e., ALS iteration process and ToA estimation. According to (29), it is observed that the number of flops required to obtain $\hat{A}$ is $O(FTKL+FKL^{2}+L^{3})$. The ALS algorithm shows an inverse relationship between iteration number and Signal-to-Noise Ratio (SNR) levels, with iterations decreasing from several hundred at low SNR to several dozen at high SNR. To improve the efficiency of the algorithm, enhanced line search [50] can be employed to accelerate the iterations for optimizing the algorithm. In addition, the computational complexity of one-dimensional line search is dominated by the calculation (32), which is required to be executed $N_{s}LK$ times, namely, computational complexity $O(N_{s}LK)$. Note that since the angle extraction process is based on the sampled covariance matrix with a low dimension, the computational complexity is ignored.

## 7. Simulation Results

This section presents numerical simulations to assess the effectiveness of the proposed tensor-based algorithm for joint channel parameter estimation and localization in 3D near-field mmWave massive MIMO-OFDM ISAC systems. In addition, we derive the CRBs for the parameters $\left\{\theta_{R,l}^{az},\theta_{R,l}^{el},\theta_{T,l}^{az},\theta_{T,l}^{el},\tau_{l}\right\}$, UT and SPs positions, which provide a lower bound of error for evaluating the performance of the proposed algorithm. The derivation process can be found in Appendix B.

The SNR is denoted as $\mathrm{SNR}={\left|\left|Y-V\right|\right|}_{F}^{2}/{\left|\left|V\right|\right|}_{F}^{2}$. The Normalized Mean Square Error (NMSE) describes the channel parameter estimation and localization performance, which is calculated as $\mathrm{NMSE}\left(\gamma \right)={\left|\left|\gamma -\hat{\gamma }\right|\right|}_{2}^{2}/{\left|\left|\gamma \right|\right|}_{2}^{2}$ with γ referring to parameter vectors $\theta_{R}^{az}=\left[\theta_{R,1}^{az},\ldots ,\theta_{R,L}^{az}\right]$, $\theta_{R}^{el}=\left[\theta_{R,1}^{el},\ldots ,\theta_{R,L}^{el}\right]$, $\theta_{T}^{az}=\left[\theta_{T,1}^{az},\ldots ,\theta_{T,L}^{az}\right]$, $\theta_{T}^{el}=\left[\theta_{T,1}^{el},\ldots ,\theta_{T,L}^{el}\right]$, $\tau =\left[\tau_{1},\ldots ,\tau_{L}\right]$ and $p_{R}$. In particular, $\mathrm{NMSE}\left(p_{l}\right)=\sum_{l=1}^{L}\|p_{l}-{\hat{p}}_{l}{\|}_{2}^{2}/\sum_{l=1}^{L}{\left\|p_{l}\right\|}_{2}^{2}$. The number of Monte Carlo trials is 600. The complex gain $\alpha_{l}$ follows a circularly symmetric complex Gaussian distribution. Other parameters are set as follows: $\bar{K}=128$, $f_{c}=30\;\mathrm{GHz}$, $N_{T}=7\times 7$, $N_{R}=7\times 7$ and $f_{s}=0.32\;\mathrm{GHz}$. Notably, in all experiments conducted, the BS functions as the sole anchor node located at a known position $p_{T}={\left[0,0,4\lambda \right]}^{T}m$, while both SPs and UT with position $p_{R}={\left[4\lambda ,4\lambda ,0\right]}^{T}m$ are treated as sensor nodes providing spatial measurement data. The UT localization depends on geometric constraints derived from SPs through reflection paths, where SP positions are randomly generated within a 4λm × 4λm × 4λm 3D space defined relative to the BS and UT. The angle and ToA parameters are generated based on the geometric relationship in the 3D environment, and the specific formulas are as follows:

(49) $$\begin{array}{cc} \theta_{R,l}^{az} & =\arctan\;2\left(y_{l}-y_{R},x_{l}-x_{R}\right)+\pi ,\\ \theta_{R,l}^{el} & =\arccos\left(\left(z_{R}-z_{l}\right)/{∥p_{l}-p_{R}∥}_{2}\right),\\ \theta_{T,l}^{az} & =\arctan\;2\left(y_{l}-y_{T},x_{l}-x_{T}\right),\\ \theta_{T,l}^{el} & =\arccos\left(\left(z_{T}-z_{l}\right)/{∥p_{l}-p_{T}∥}_{2}\right),\\ \tau_{l} & ={∥p_{l}-p_{R}∥}_{2}/c+{∥p_{T}-p_{l}∥}_{2}/c. \end{array}$$

Note that this work operates under the quasi-static assumption where the mobile velocity of UT is sufficiently low to ensure negligible Doppler frequency shift and time-invariant channel parameters throughout the processing interval. In the future, we will study channel estimation and target localization in high-speed mobile scenarios, and establish high-order tensor models to capture the time-varying characteristics of the channel. To focus the research on tensor signal processing, the simulation experiments in this paper are conducted under ideal environmental settings for parameter estimation and target localization. For non-Gaussian noise, previous work [51] has achieved the phase noise estimation at both the transmitter and receiver. The proposed algorithm in the paper adopts assumptions including perfect transceiver synchronization and ideal hardware components (neglecting nonlinear power amplifier and I/Q imbalance) to enable fair performance comparison under controlled conditions, as these impairments can be effectively compensated by dedicated modules in practical implementations. We will further consider non-ideal conditions such as hardware defects and non-Gaussian noise in subsequent research.

**Figure 4:**
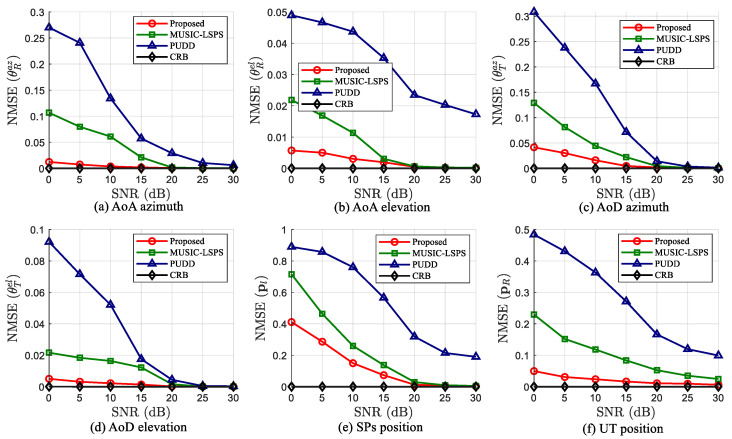
Parameter estimation and target localization performance of different algorithms versus SNR, when F=T=50, K=10 and L=3.

In the first experiment, we compare the parameter estimation and target localization performance of the proposed tensor-based algorithm with two existing algorithms. Additionally, the CRBs of channel parameters and target localization are used as a lower bound of error for reference. Figure 4 shows the NMSE curves of the estimated angle parameters and positions versus SNR at F=T=50, L=3, K=10 and SNR = 0–30 dB. It can be observed that the proposed algorithm outperforms the other two algorithms in terms of ISAC performance, especially under low SNR conditions, and its performance is close to the theoretical error lower limit. For example, the gap between the proposed algorithm and MUSIC-LSPS algorithm reaches 90.8% for AoA azimuth, when the SNR = 5 dB. Since the MUSIC-LSPS algorithm is a spectral peak search algorithm on sampling response vectors, it has inherent grid errors. In addition, the PUDD algorithm performs least squares operation on the basis of estimated distance difference, which leads to error accumulation. In contrast, by exploiting the inherent structure of the received signal, the proposed algorithm enables direct angle estimation through factor matrices, eliminating both the need for sampling vectors and the accumulation of approximation errors. From the above analysis, it can be seen that the proposed algorithm performs optimally in terms of channel parameter estimation and localization, and is closest to the CRBs. This capability highlights the potential of the proposed algorithm for practical ISAC applications.

**Figure 5:**
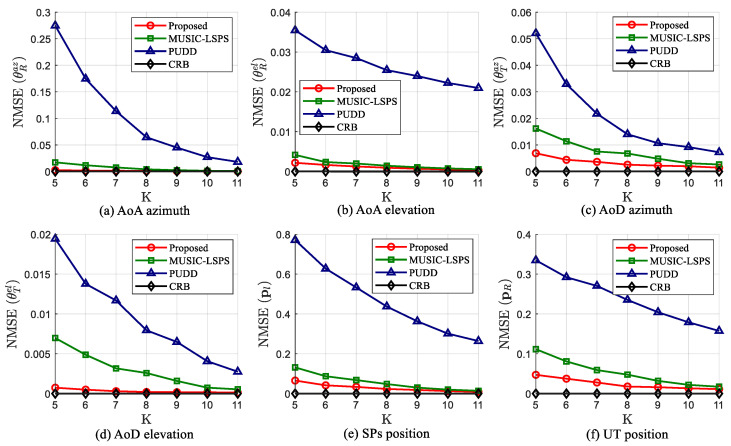
Parameter estimation and target localization performance of different algorithms versus *K*, when F=T=50, SNR = 20 dB and L=3.

In the second experiment, we investigate the impact of the number of subcarriers *K* on channel parameter estimation accuracy and the localization performance of both SPs and UT for our proposed algorithm compared to existing methods. Figure 5 describes the NMSE curves of the angle and position versus *K*, when F=T=50, L=3 and SNR = 20 dB. Due to the expansion of the tensor dimension, the estimation accuracy of the factor matrices is improved, which further enhances the accuracy of parameter estimation. Therefore, the increase in the number of subcarriers will lead to improvements in channel parameter estimation and localization performance. As described in the previous paragraph, the proposed algorithm does not have the drawbacks of the other two algorithms, which holds true under different subcarrier configurations. As illustrated in Figure 5, the proposed algorithm obtains best estimation and localization accuracy for *K* = 5–11, followed by the MUSIC-LSPS algorithm, and the PUDD algorithm is the worst. Unlike existing methods whose accuracy degrades significantly with reduced subcarriers, the proposed algorithm consistently maintains optimal estimation performance across the entire subcarrier range. Furthermore, when *K* = 5–11, the proposed algorithm is the closest to the CRB, which to some extent indicates that the proposed algorithm has certain robustness.

**Figure 6:**
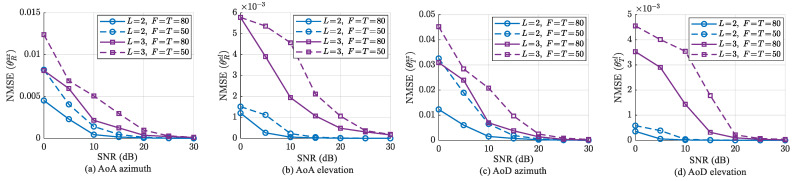
NMSE of angle parameters versus SNR when L=2,3, F=T={50,80} and K=8.

**Figure 7:**
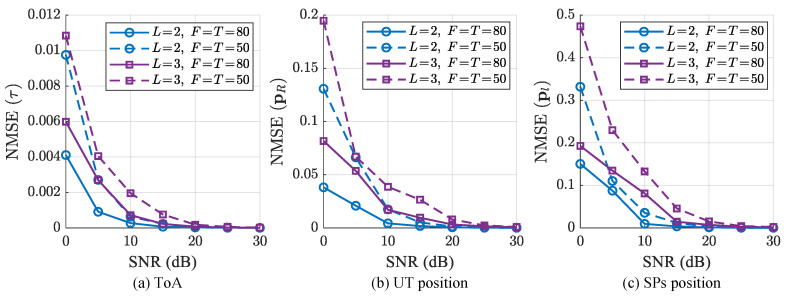
NMSE of ToA and localization versus SNR when L=2,3, F=T={50,80} and K=8.

**Figure 8:**
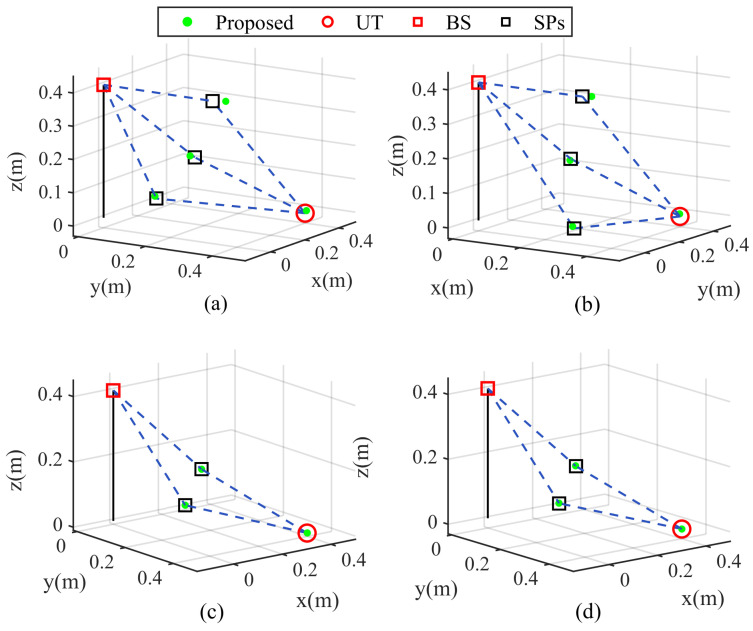
Three-dimensional localization visualization at K=8: (**a**) L=3, F=T=50 (**b**) L=3, F=T=80 (**c**) L=2, F=T=50 (**d**) L=2, F=T=80.

In the third simulation, we analyze the impact of varying the number of paths and time frames on the ISAC performance of proposed algorithm. Figure 6 shows the NMSE curves versus SNR in case 1 (L=2,F=T=80), case 2 (L=2,F=T=50), case 3 (L=3,F=T=80) and case 4 (L=3,F=T=50), where K=8. From Figure 6, we observe that channel parameter estimation precision improves with more time frames. Moreover, it is seen that the NMSE performance demonstrate a decreasing trend with an increasing number of paths. The accuracy of localization and ToA estimation for the proposed algorithm under these configurations are shown in Figure 7. Moreover, Figure 8 indicates the 3D visualization of the SPs and UT localization. Obviously, the localization accuracy in Figure 8 is consistent with the trends shown in Figure 7 where the increasing F,T and decreasing *L* improve localization performance. The error data in Figure 8 are presented in Table 2, where SNR = 5 dB. Specifically, according to formula (47), the localization of SP depends on all parameters and the position of UT. The propagation of errors leads to a relatively larger error for the localization of SP compared to that of UT. As the number of paths increases, channel sparsity diminishes, which adversely affects channel parameter estimation and leads to a certain degree of degradation in localization performance. In addition, even though more sensor nodes are available as *L* increases, the reduction in channel sparsity decreases the accuracy of parameter estimation, thereby lowering the localization accuracy of UT. Moreover, the localization accuracy improves with increasing *F* and *T*, which reflects the growth in transmitted data volume. Therefore, the proposed algorithm can effectively achieve simultaneous localization and estimation in ISAC scenarios.

## 8. Conclusions

In this paper, a tensor-based channel parameter estimation and localization algorithm has been proposed for the near-field mmWave massive MIMO-OFDM ISAC system, which combines CP decomposition and the internal structure of factor matrices. The proposed algorithm fully exploits the low-rank property of tensors and the sparsity of mmWave channels. The simulation results show that the proposed tensor-based algorithm achieves superior ISAC performance for IoT networks in terms of channel parameter estimation and localization compared with existing algorithms, especially at low SNR, and is closer to the rigorously derived CRBs. Specifically, the proposed algorithm improves the accuracy by 79.8% and 38.3% compared to the suboptimal algorithm for the UT and SP localization when SNR = 5 dB, respectively.

The spatial-wideband effect emerges when propagation delays across large antenna arrays cause significant phase variations between antennas, making conventional MIMO models inadequate as delays approach or exceed symbol durations. Future work will tackle channel estimation, symbol detection and target localization in spatial-wideband massive MIMO systems by advancing higher-order tensor methods for multi-dimensional signal processing. Furthermore, building upon our previous discussion, we will extend this research to investigate time-varying channel estimation under non-ideal system configurations.

## Figures and Tables

**Table 1:** Summary of symbolic representations.

Symbol	Definition
$N_{T}/N_{R}$	Number of antennas at BS/UT
$N_{T}^{y}/N_{R}^{y}$	Number of horizontal antennas at BS/UT
$N_{T}^{z}/N_{R}^{z}$	Number of vertical antennas at BS/UT
$M_{T}/M_{R}$	Number of RF chains at BS/UT
$p_{T}/p_{R}/p_{l}$	Position coordinates of BS/UT/SP
$D_{T}/D_{R}$	Near-field range of BS/UT
$\bar{K}$	OFDM subcarriers
*K*	Selected subcarriers for ISAC
*T*	Number of time frames
*F*	Number of sub-frames per time frame
*S*	Number of pilot symbols
$F_{\mathrm{RF},t}$	RF precoding at time frame *t*
$F_{k,t}$	Digital precoding for subcarrier *k* at time frame *t*
$s_{k,t}$	Pilot symbol vector for subcarrier *k* at time frame *t*
$f_{t}$	The *t*-th column of hybrid precoder matrix F
$w_{f}$	The *f*-th column of hybrid combiner matrix W
$H_{k}$	Near-field channel for subcarrier *k*
$Y_{k}$	Received signal for subcarrier *k*
*L*	Number of paths
$\tau_{l}$	Time delay for path *l*
$\alpha_{l}$	Complex gain for path *l*
$\theta_{R,l}^{az}/\theta_{T,l}^{az}$	Azimuth AoA/AoD at UT/BS
$\theta_{R,l}^{el}/\theta_{T,l}^{el}$	Elevation AoA/AoD at UT/BS
$d_{R,l}^{c}/d_{T,l}^{c}$	Distance from the SP *l* to the centers of UT/BS
$d_{R,l}^{n_{R}}/d_{T,l}^{n_{T}}$	Distance from the SP *l* to the $n_{R}$-th/$n_{T}$-th antenna of UT/BS
$a_{R,l}/b_{T,l}$	Response vector at UT/BS

**Table 2:** NMSE of localization estimation under different cases.

Targets	Case 1	Case 2	Case 3	Case 4
UT	0.0208	0.0663	0.0536	0.0670
SPs	0.0873	0.1102	0.1344	0.2296

## Data Availability

The data that support the findings of this study are available from the corresponding author upon reasonable request.

## Appendix A

Suppose that the error matrix corresponding to the factor matrix C can be expressed as $E_{c}=\left[e_{1},\ldots ,e_{L}\right]\in C^{K\times L}$, and we have $\hat{C}=CD_{c}\Pi +E_{c}$, where $D_{c}$ and Π are the non-singular diagonal matrix and permutation matrix, respectively. Therefore, the relationship between the error vector $e_{l}$ and $c_{l}$ can be given by $c_{l}=\omega_{l}c_{l}+e_{l}$, where $\omega_{l}$ refers to the scaling factor. Given that the error vector $e_{l}$ follows circularly symmetric complex Gaussian distributions with zero mean and covariance $\sigma^{2}I_{K}$, we obtain

(A1) $$\begin{array}{cc} P(e_{l}) & =\frac{1}{\pi^{K}\det\left(\sigma^{2}I_{K}\right)}\exp\left(-{e_{l}}^{H}{\left(\sigma^{2}I_{K}\right)}^{-1}e_{l}\right)\\ \; & =\frac{1}{\pi^{K}{\left(\sigma^{2}\right)}^{K}}\exp(-\frac{1}{\sigma^{2}}\left|\right|e_{l}{\left|\right|}_{F}^{2}), \end{array}$$

thus, the likelihood function regarding $\omega_{l}$ and $\tau_{l}$ is denoted as

(A2) $$\begin{array}{c} \Upsilon \left(\omega_{l},\tau_{l}\right)=-K\ln\left(\pi \sigma^{2}\right)-\frac{1}{\sigma^{2}}\left|\right|{\hat{c}}_{l}-\omega_{l}c_{l}\left(\tau_{l}\right){\left|\right|}_{F}^{2} \end{array}.$$

It can be seen from (A2) that maximizing the log-likelihood function is equivalent to minimizing the squared errors, i.e.,

(A3) $$J\left(\omega_{l},\tau_{l}\right)={∥{\hat{c}}_{l}-\omega_{l}c_{l}\left(\tau_{l}\right)∥}_{F}^{2}.$$

Differentiating function $J\left(\omega_{l},\tau_{l}\right)$ with respect to $\omega_{l}^{*}$ yields

(A4) $$\frac{\partial J}{\partial \omega_{l}^{*}}=\frac{\partial }{\partial \omega_{l}^{*}}{\left({\hat{c}}_{l}-\omega_{l}c_{l}\left(\tau_{l}\right)\right)}^{H}\left({\hat{c}}_{l}-\omega_{l}c_{l}\left(\tau_{l}\right)\right)=0,$$

whose solution is

(A5) $$\omega_{l}=\frac{c_{l}^{H}\left(\tau_{l}\right){\hat{c}}_{l}}{{∥c_{l}\left(\tau_{l}\right)∥}_{F}^{2}}.$$

Substituting (A5) into (A3), we can obtain

(A6) $$J\left(\tau_{l}\right)={∥{\hat{c}}_{l}-\frac{c_{l}^{H}\left(\tau_{l}\right){\hat{c}}_{l}}{{∥c_{l}\left(\tau_{l}\right)∥}_{F}^{2}}c_{l}\left(\tau_{l}\right)∥}_{F}^{2}={∥{\hat{c}}_{l}∥}_{F}^{2}-\frac{{\left|c_{l}^{H}\left(\tau_{l}\right){\hat{c}}_{l}\right|}^{2}}{{∥c_{l}\left(\tau_{l}\right)∥}_{F}^{2}}.$$

Therefore, the estimation of $\tau_{l}$ can be achieved by solving the following problem

(A7) $$\tau_{l}=\underset{\tau_{l}}{\arg\max}\frac{{\left|c_{l}^{H}\left(\tau_{l}\right){\hat{c}}_{l}\right|}^{2}}{{∥c_{l}\left(\tau_{l}\right)∥}_{F}^{2}}.$$

## Appendix B

### Appendix B.1. CRBs of Channel Parameters

The CRB is considered the minimum achievable variance for an unbiased estimator, representing the ultimate asymptotic performance limit in estimation theory. Thus, we derive the CRBs of the parameter vector $\varpi =\left[\theta_{R}^{az},\theta_{R}^{el},\theta_{T}^{az},\theta_{T}^{el},\tau \right]$ in the subsection. Since the entries of the noise tensor V are independent and identically distributed circularly symmetric Gaussian random variables with zero mean and variance $\varsigma^{2}$, the likelihood function for the parameter vector can be expressed as

(A8) $$\begin{array}{cc} F\left(\varpi \right)= & -TFK\ln\left(\pi \varsigma^{2}\right)-\frac{1}{\varsigma^{2}}{∥{\left[Y\right]}_{\left(1\right)}-\left(C⊙B\right)A^{T}∥}_{F}^{2}\\ = & -TFK\ln\left(\pi \varsigma^{2}\right)-\frac{1}{\varsigma^{2}}{∥{\left[Y\right]}_{\left(2\right)}-\left(C⊙A\right)B^{T}∥}_{F}^{2}\\ = & -TFK\ln\left(\pi \varsigma^{2}\right)-\frac{1}{\varsigma^{2}}{∥{\left[Y\right]}_{\left(3\right)}-\left(B⊙A\right)C^{T}∥}_{F}^{2}. \end{array}$$

The Fisher Information Matrix (FIM) corresponding to ϖ can be given by

(A9) $$\begin{array}{cc} \Omega \left(\varpi \right) & =E\left\{{\left(\frac{\partial F\left(\varpi \right)}{\partial \varpi }\right)}^{H}\left(\frac{\partial F\left(\varpi \right)}{\partial \varpi }\right)\right\} \end{array}.$$

For simplicity, we take the partial derivative of $F\left(\varpi \right)$ with respect to $\theta_{R,l}^{az}$ as an example, which is computed as

(A10) $$\frac{\partial F\left(\varpi \right)}{\partial \theta_{R,l}^{az}}=tr\left\{{\left(\frac{\partial F\left(\varpi \right)}{\partial A}\right)}^{T}\frac{\partial A}{\partial \theta_{R,l}^{az}}+{\left(\frac{\partial F\left(\varpi \right)}{\partial A^{*}}\right)}^{T}\frac{\partial A^{*}}{\partial \theta_{R,l}^{az}}\right\},$$

where

(A11) $$\begin{array}{cc} \frac{\partial F\left(\varpi \right)}{\partial A}= & \frac{1}{\varsigma^{2}}{\left({\left[Y\right]}_{\left(1\right)}-\left(C⊙B\right)A^{T}\right)}^{H}\left(C⊙B\right),\\ \frac{\partial F\left(\varpi \right)}{\partial A^{*}}= & \frac{1}{\varsigma^{2}}{\left({\left[Y\right]}_{\left(1\right)}-\left(C⊙B\right)A^{T}\right)}^{T}{\left(C⊙B\right)}^{*},\\ \frac{\partial A}{\partial \theta_{R,l}^{az}}= & [0,\ldots ,{\overset{⌢}{a}}_{l}^{az},\ldots ,0],\\ {\overset{⌢}{a}}_{l}^{az}= & W^{T}\Lambda_{A}^{az}a_{R,l},\\ \Lambda_{A}^{az}= & \frac{j2\pi dd_{R,l}^{c}\cos\theta_{R,l}^{az}\sin\theta_{R,l}^{el}}{\lambda d_{R,l}^{n_{R}}}diag\left(\Phi_{1},\Phi_{2},\ldots ,\Phi_{N_{R}^{z}}\right),\\ \Phi_{n_{R}^{y}}= & diag(-\left(N_{R}^{y}-1\right)/2,\ldots ,0,\ldots ,\left(N_{R}^{y}-1\right)/2). \end{array}$$

Therefore, we can obtain

(A12) ∂Fϖ∂θR,laz=elT1ς2(C⊙B)T([Y](1)−(C⊙B)AT)*a⌢laz+elT1ς2(C⊙B)H([Y](1)−(C⊙B)AT)a⌢laz*=2ReelT1ς2C⊙BT[Y](1)−C⊙BAT*A⌢azel,

where $e_{l}$ is a vector whose *l*-th element is not zero. Then, the first partial derivatives of the likelihood function F with respect to other parameters can be computed as

(A13) ∂F(ϖ)∂θR,lel=2ReelT1ς2C⊙BT[Y](1)−C⊙BAT*A⌢elel,∂F(ϖ)∂θT,laz=2ReelT1ς2(C⊙A)T([Y](2)−(C⊙A)BT)*B⌢azel,∂F(ϖ)∂θT,lel=2ReelT1ς2(C⊙A)T([Y](2)−(C⊙A)BT)*B⌢elel,∂F(ϖ)∂τl=2ReelT1ς2B⊙AT[Y](3)−B⊙ACT*C⌢τel,

where ${\overset{⌢}{A}}_{az(el)}=\left[{\overset{⌢}{a}}_{1}^{az(el)},\ldots ,{\overset{⌢}{a}}_{L}^{az(el)}\right]$, ${\overset{⌢}{B}}_{az(el)}=\left[{\overset{⌢}{b}}_{1}^{az(el)},\ldots ,{\overset{⌢}{b}}_{L}^{az(el)}\right],$${\overset{⌢}{C}}_{\tau }=\left[{\overset{⌢}{c}}_{1}^{\tau },\ldots ,{\overset{⌢}{c}}_{L}^{\tau }\right]$,

(A14) $$\begin{array}{cc} {\overset{⌢}{a}}_{l}^{el}= & W^{T}\Lambda_{A}^{el}a_{R,l},\\ \Lambda_{A}^{el}= & \frac{j2\pi dd_{R,l}^{c}}{\lambda d_{R,l}^{n_{R}}}(\sin\theta_{R,l}^{az}\cos\theta_{R,l}^{el}diag\left(\Phi_{1},\Phi_{2},\ldots ,\Phi_{N_{R}^{z}}\right)\\ + & \sin\theta_{R,l}^{el}diag\left(-\left(N_{R}^{y}-1\right)/2,\ldots ,0,\ldots ,\left(N_{R}^{y}-1\right)/2\right)⊗I_{N_{R}^{y}}),\\ {\overset{⌢}{c}}_{l}^{\tau }= & -j2\pi f_{s}/\bar{K}diag\left(0,1,\ldots ,K-1\right)c_{l}. \end{array}$$

Note that the partial derivative of the matrix B is similar to the matrix A, which will not be shown in detail. Furthermore, the $\left(l_{1},l_{2}\right)$-th element of

(A15) $$E\left\{{\left(\frac{\partial F\left(\varpi \right)}{\partial \theta_{R}^{az}}\right)}^{H}\left(\frac{\partial F\left(\varpi \right)}{\partial \theta_{R}^{az}}\right)\right\}$$

can be expressed as

(A16) E∂Fϖ∂θR,l1az*∂Fϖ∂θR,l2az=4ERe{el1TNAazel1}Re{el2TNAazel2}=E(NAaz(l1,l1)+NAaz(l1,l1)*)(NAaz(l2,l2)+NAaz(l2,l2)*),

where

(A17) $$N_{A}^{az}=\frac{1}{\varsigma^{2}}{\left(C⊙B\right)}^{T}{\left({\left[Y\right]}_{\left(1\right)}-\left(C⊙B\right)A^{T}\right)}^{*}{\overset{⌢}{A}}_{az}=\frac{1}{\varsigma^{2}}{\left(C⊙B\right)}^{T}{\left[V\right]}_{\left(1\right)}^{*}{\overset{⌢}{A}}_{az}.$$

We define $n_{A}^{az}=\mathrm{vec}\left(N_{A}^{az}\right)$ as the vector expansion expression for $N_{A}^{az}$, which can be given by

(A18) $$\mathrm{vec}\left(N_{A}^{az}\right)=\frac{1}{\varsigma^{2}}\left({\overset{⌢}{A}}_{az}^{T}⊗{\left(C⊙B\right)}^{T}\right)\mathrm{vec}\left({\left[V\right]}_{\left(1\right)}^{*}\right),$$

The above equation is equivalent to a linear transformation of $\mathrm{vec}({\left[V\right]}_{\left(1\right)}^{*})$; therefore, the vector $n_{A}^{az}$ also follows the same distribution as $\mathrm{vec}({\left[V\right]}_{\left(1\right)}^{*})$. Its covariance matrix $R_{n_{A}^{az}}\in C^{L^{2}\times L^{2}}$ and second-order moment $M_{n_{A}^{az}}\in C^{L^{2}\times L^{2}}$ are expressed, respectively, as

(A19) $$R_{n_{A}^{az}}=E\left[\left(n_{A}^{az}\right){\left(n_{A}^{az}\right)}^{H}\right]=\frac{1}{\varsigma^{2}}\left({\overset{⌢}{A}}_{az}^{T}⊗{\left(C⊙B\right)}^{T}\right)\left({\overset{⌢}{A}}_{az}^{*}⊗{\left(C⊙B\right)}^{*}\right),$$

and

(A20) $$R_{n_{A}^{az}}=E\left[\left(n_{A}^{az}\right){\left(n_{A}^{az}\right)}^{T}\right]=0.$$

Since only the cross-conjugate term is not 0 in (A16), we obtain

(A21) E∂Fϖ∂θR,l1az*∂Fϖ∂θR,l2az=2Re{RnAaz(p,q)},

where $p≜L\left(l_{1}-1\right)+l_{1}$ and $q≜L\left(l_{2}-1\right)+l_{2}$. Similarly, the covariance for other parameters can be given by

(A22) E∂Fϖ∂θR,l1el*∂Fϖ∂θR,l2el=2Re{RnAel(p,q)},E∂Fϖ∂θT,l1az*∂Fϖ∂θT,l2az=2Re{RnBaz(p,q)},E∂Fϖ∂θT,l1el*∂Fϖ∂θT,l2el=2Re{RnBel(p,q)},E∂Fϖ∂τl1*∂Fϖ∂τl2=2Re{RnCτ(p,q)},

where

(A23) $$\begin{array}{cc} R_{n_{A}^{az}} & =\frac{1}{\varsigma^{2}}\left({\overset{⌢}{A}}_{az}^{T}⊗{\left(C⊙B\right)}^{T}\right)\left({\overset{⌢}{A}}_{az}^{*}⊗{\left(C⊙B\right)}^{*}\right),\\ R_{n_{A}^{el}} & =\frac{1}{\varsigma^{2}}\left({\overset{⌢}{A}}_{el}^{T}⊗{\left(C⊙B\right)}^{T}\right)\left({\overset{⌢}{A}}_{el}^{*}⊗{\left(C⊙B\right)}^{*}\right),\\ R_{n_{B}^{az}} & =\frac{1}{\varsigma^{2}}\left({\overset{⌢}{B}}_{az}^{T}⊗{\left(C⊙A\right)}^{T}\right)\left({\overset{⌢}{B}}_{az}^{*}⊗{\left(C⊙A\right)}^{*}\right),\\ R_{n_{B}^{el}} & =\frac{1}{\varsigma^{2}}\left({\overset{⌢}{B}}_{el}^{T}⊗{\left(C⊙A\right)}^{T}\right)\left({\overset{⌢}{B}}_{el}^{*}⊗{\left(C⊙A\right)}^{*}\right),\\ R_{n_{C}^{\tau }} & =\frac{1}{\varsigma^{2}}\left({\overset{⌢}{C}}_{\tau }^{T}⊗{\left(B⊙A\right)}^{T}\right)\left({\overset{⌢}{C}}_{\tau }^{*}⊗{\left(B⊙A\right)}^{*}\right). \end{array}$$

Considering the other non-pivot entries in $\Omega \left(\varpi \right)$, such as

(A24) E∂Fϖ∂θR,l1az*∂Fϖ∂θT,l2az=4ERe{el1TNAazel1}Re{el2TNBazel2}=E(NAaz(l1,l1)+NAaz(l1,l1)*)(NBaz(l2,l2)+NBaz(l2,l2)*)=2Re{RnAaz,nBaz(p,q)},

in which

(A25) $$R_{n_{A}^{az},n_{B}^{az}}=E\left[\left(n_{A}^{az}\right){\left(n_{B}^{az}\right)}^{H}\right]=\frac{1}{\varsigma^{4}}\left({\overset{⌢}{A}}_{az}^{T}⊗{\left(C⊙B\right)}^{T}\right)R_{v_{1},v_{2}}\left({\overset{⌢}{B}}_{az}^{*}⊗{\left(C⊙A\right)}^{*}\right).$$

Similarly, the following covariance formulas can be obtained

(A26) $$\begin{array}{cc} R_{n_{A}^{az},n_{A}^{el}} & =\frac{1}{\varsigma^{2}}\left({\overset{⌢}{A}}_{az}^{T}⊗{\left(C⊙B\right)}^{T}\right)\left({\overset{⌢}{A}}_{el}^{*}⊗{\left(C⊙B\right)}^{*}\right),\\ R_{n_{A}^{az},n_{B}^{el}} & =\frac{1}{\varsigma^{4}}\left({\overset{⌢}{A}}_{az}^{T}⊗{\left(C⊙B\right)}^{T}\right)R_{v_{1},v_{2}}\left({\overset{⌢}{B}}_{el}^{*}⊗{\left(C⊙A\right)}^{*}\right),\\ R_{n_{A}^{az},n_{C}^{\tau }} & =\frac{1}{\varsigma^{4}}(\left({\overset{⌢}{A}}_{az}^{T}⊗{\left(C⊙B\right)}^{T}\right)R_{v_{1},v_{3}}\left({\overset{⌢}{C}}_{\tau }^{*}⊗{\left(B⊙A\right)}^{*}\right),\\ R_{n_{B}^{az},n_{C}^{\tau }} & =\frac{1}{\varsigma^{4}}(\left({\overset{⌢}{B}}_{az}^{T}⊗{\left(C⊙A\right)}^{T}\right)R_{v_{2},v_{3}}\left({\overset{⌢}{C}}_{\tau }^{*}⊗{\left(B⊙A\right)}^{*}\right), \end{array}$$

where

(A27) $$\begin{array}{cc} R_{v_{1},v_{2}} & =E\{vec\left({\left[V\right]}_{\left(1\right)}^{*}\right)vec{\left({\left[V\right]}_{\left(2\right)}\right)}^{T}\},\\ R_{v_{1},v_{3}} & =E\{vec\left({\left[V\right]}_{\left(1\right)}^{*}\right)vec{\left({\left[V\right]}_{\left(3\right)}\right)}^{T}\},\\ R_{v_{2},v_{3}} & =E\{vec\left({\left[V\right]}_{\left(2\right)}^{*}\right)vec{\left({\left[V\right]}_{\left(3\right)}\right)}^{T}\}. \end{array}$$

Note that the covariance matrices for the other parameters follow a similar form to the (A26) and thus are omitted for brevity. Obviously, the $\left(f,t,k\right)$-th element of the noise tensor corresponds to the $\left(t+(k-1)T,f\right)$-th, $\left(f+(k-1)F,t\right)$-th and $\left(f+(t-1)F,k\right)$-th elements of ${\left[V\right]}_{\left(1\right)}$, ${\left[V\right]}_{\left(2\right)}$ and ${\left[V\right]}_{\left(3\right)}$, respectively. Further, leveraging the index relationships between the modal unfolding $\mathrm{vec}(V_{\left(1\right)})$, $\mathrm{vec}(V_{\left(2\right)})$, $\mathrm{vec}(V_{\left(2\right)})$ and the tensor V, along with the property of the independent and identically distributed tensor V, we derive that the indexes $\left\{\left(p,q\right)|R_{v_{1},v_{2}}\left(p,q\right)\neq 0\right\}$ can be expressed as

(A28) $$\left\{\begin{array}{cc} \; & p=t+(k-1)T+(f-1)TK\\ \; & q=f+(t-1)F+(k-1)TF \end{array}\right..$$

Similarly, the non-zero element indexes for $R_{v_{1},v_{3}}$ and $R_{v_{2},v_{3}}$, respectively, belong to

(A29) $$\left\{\begin{array}{cc} \; & p=t+(k-1)T+(f-1)TK\\ \; & q=f+(t-1)F+(k-1)FT \end{array}\right.,$$

and

(A30) $$\left\{\begin{array}{cc} \; & p=f+(k-1)F+(t-1)FK\\ \; & q=f+(t-1)F+(k-1)FT \end{array}\right..$$

Based on the above analysis, the CRBs of parameter vector ϖ can be obtained by

(A31) $$\mathrm{CRB}\left(\varpi \right)=\Omega^{-1}\left(\varpi \right).$$

### Appendix B.2. CRBs of UT and SPs Positions

Utilizing the (A31), the CRBs for the UT and SPs positions can be obtained based on the FIM Ω(ϖ) [15]. The FIMs of UE and SPs can be given as

(A32) $$\begin{array}{cc} \Omega_{UT} & =\left(\nabla_{p_{R}}\varpi \right)\Omega \left(\varpi \right){\left(\nabla_{p_{R}}\varpi \right)}^{H},\\ \Omega_{SP} & =\left(\nabla_{p_{l}}\varpi \right)\Omega \left(\varpi \right){\left(\nabla_{p_{l}}\varpi \right)}^{H}, \end{array}$$

where $\nabla_{p_{R}}\varpi$ is denoted as the partial derivative matrix with respect to $p_{R}$, whose size is 3×5L. Next, taking $\nabla_{p_{R}}\varpi$ as an example, in detail

(A33) $$\nabla_{p_{R}}\varpi =\left[\begin{array}{ccccc} \nabla_{x_{R}}\theta_{R}^{az} & \nabla_{x_{R}}\theta_{R}^{el} & \nabla_{x_{R}}\theta_{T}^{az} & \nabla_{x_{R}}\theta_{T}^{el} & \nabla_{x_{R}}\tau \\ \nabla_{y_{R}}\theta_{R}^{az} & \nabla_{y_{R}}\theta_{R}^{el} & \nabla_{y_{R}}\theta_{T}^{az} & \nabla_{y_{R}}\theta_{T}^{el} & \nabla_{y_{R}}\tau \\ \nabla_{z_{R}}\theta_{R}^{az} & \nabla_{z_{R}}\theta_{R}^{el} & \nabla_{z_{R}}\theta_{T}^{az} & \nabla_{z_{R}}\theta_{T}^{el} & \nabla_{z_{R}}\tau \end{array}\right],$$

where

(A34) $$\begin{array}{cc} \nabla_{x_{R}}\theta_{T}^{az(el)} & =\nabla_{y_{R}}\theta_{T}^{az(el)}=\nabla_{z_{R}}\theta_{T}^{az(el)}=0_{1\times L},\\ \nabla_{x_{R}}\theta_{R}^{az} & =\frac{y_{R}-y_{l}}{{\left(x_{l}-x_{R}\right)}^{2}+{\left(y_{l}-y_{R}\right)}^{2}},\\ \nabla_{y_{R}}\theta_{R}^{az} & =\frac{x_{l}-x_{R}}{{\left(x_{l}-x_{R}\right)}^{2}+{\left(y_{l}-y_{R}\right)}^{2}},\\ \nabla_{z_{R}}\theta_{R}^{az} & =0,\\ \nabla_{x_{R}}\theta_{R}^{el} & =\frac{\left(z_{R}-z_{l}\right)\left(x_{R}-x_{l}\right)}{\|p_{l}-p_{R}\|\sqrt{\|p_{l}-p_{R}{\|}^{2}-{\left(z_{R}-z_{l}\right)}^{2}}},\\ \nabla_{y_{R}}\theta_{R}^{el} & =\frac{\left(z_{R}-z_{l}\right)\left(y_{R}-y_{l}\right)}{\|p_{l}-p_{R}\|\sqrt{\|p_{l}-p_{R}{\|}^{2}-{\left(z_{R}-z_{l}\right)}^{2}}},\\ \nabla_{z_{R}}\theta_{R}^{el} & =\frac{-[{\left(x_{l}-x_{R}\right)}^{2}+{\left(y_{l}-y_{R}\right)}^{2}]}{\|p_{l}-p_{R}\|\sqrt{\|p_{l}-p_{R}{\|}^{2}-{\left(z_{R}-z_{l}\right)}^{2}}},\\ \nabla_{{x(y,z)}_{R}}\tau & =\frac{x{\left(y,z\right)}_{R}-x{\left(y,z\right)}_{l}}{c\|p_{l}-p_{R}\|}. \end{array}$$

The FIM of SPs can be computed similarly. Finally, the CRBs of UE and SPs positions can be obtained by

(A35) $$\mathrm{CRB}\left(p_{R}\right)=\Omega_{UT}^{-1},\mathrm{CRB}\left(p_{l}\right)=\Omega_{SP}^{-1}.$$
